# Applications of peptides in nanosystems for diagnosing and managing bacterial sepsis

**DOI:** 10.1186/s12929-024-01029-2

**Published:** 2024-04-19

**Authors:** Mohammed A. Gafar, Calvin A. Omolo, Eman Elhassan, Usri H. Ibrahim, Thirumala Govender

**Affiliations:** 1 Discipline of Pharmaceutical Sciences, College of Health Sciences, University of KwaZulu-Natal, Private Bag X54001, Durban, South Africa; 2 Department of Pharmaceutics, Faculty of Pharmacy, University of Khartoum, P.O. Box 1996, Khartoum, Sudan; 3 Department of Pharmaceutics and Pharmacy Practice, School of Pharmacy and Health Sciences, United States International University-Africa, P. O. Box 14634-00800, Nairobi, Kenya; 4 Discipline of Human Physiology, School of Laboratory Medicine and Medical Sciences, College of Health Sciences, University of KwaZulu-Natal, Durban, South Africa

**Keywords:** Peptide, Sepsis, Nanosystem, Sepsis diagnosis, Sepsis management, Drug delivery

## Abstract

Sepsis represents a critical medical condition stemming from an imbalanced host immune response to infections, which is linked to a significant burden of disease. Despite substantial efforts in laboratory and clinical research, sepsis remains a prominent contributor to mortality worldwide. Nanotechnology presents innovative opportunities for the advancement of sepsis diagnosis and treatment. Due to their unique properties, including diversity, ease of synthesis, biocompatibility, high specificity, and excellent pharmacological efficacy, peptides hold great potential as part of nanotechnology approaches against sepsis. Herein, we present a comprehensive and up-to-date review of the applications of peptides in nanosystems for combating sepsis, with the potential to expedite diagnosis and enhance management outcomes. Firstly, sepsis pathophysiology, antisepsis drug targets, current modalities in management and diagnosis with their limitations, and the potential of peptides to advance the diagnosis and management of sepsis have been adequately addressed. The applications have been organized into diagnostic or managing applications, with the last one being further sub-organized into nano-delivered bioactive peptides with antimicrobial or anti-inflammatory activity, peptides as targeting moieties on the surface of nanosystems against sepsis, and peptides as nanocarriers for antisepsis agents. The studies have been grouped thematically and discussed, emphasizing the constructed nanosystem, physicochemical properties, and peptide-imparted enhancement in diagnostic and therapeutic efficacy. The strengths, limitations, and research gaps in each section have been elaborated. Finally, current challenges and potential future paths to enhance the use of peptides in nanosystems for combating sepsis have been deliberately spotlighted. This review reaffirms peptides' potential as promising biomaterials within nanotechnology strategies aimed at improving sepsis diagnosis and management.

**Graphical Abstract:**

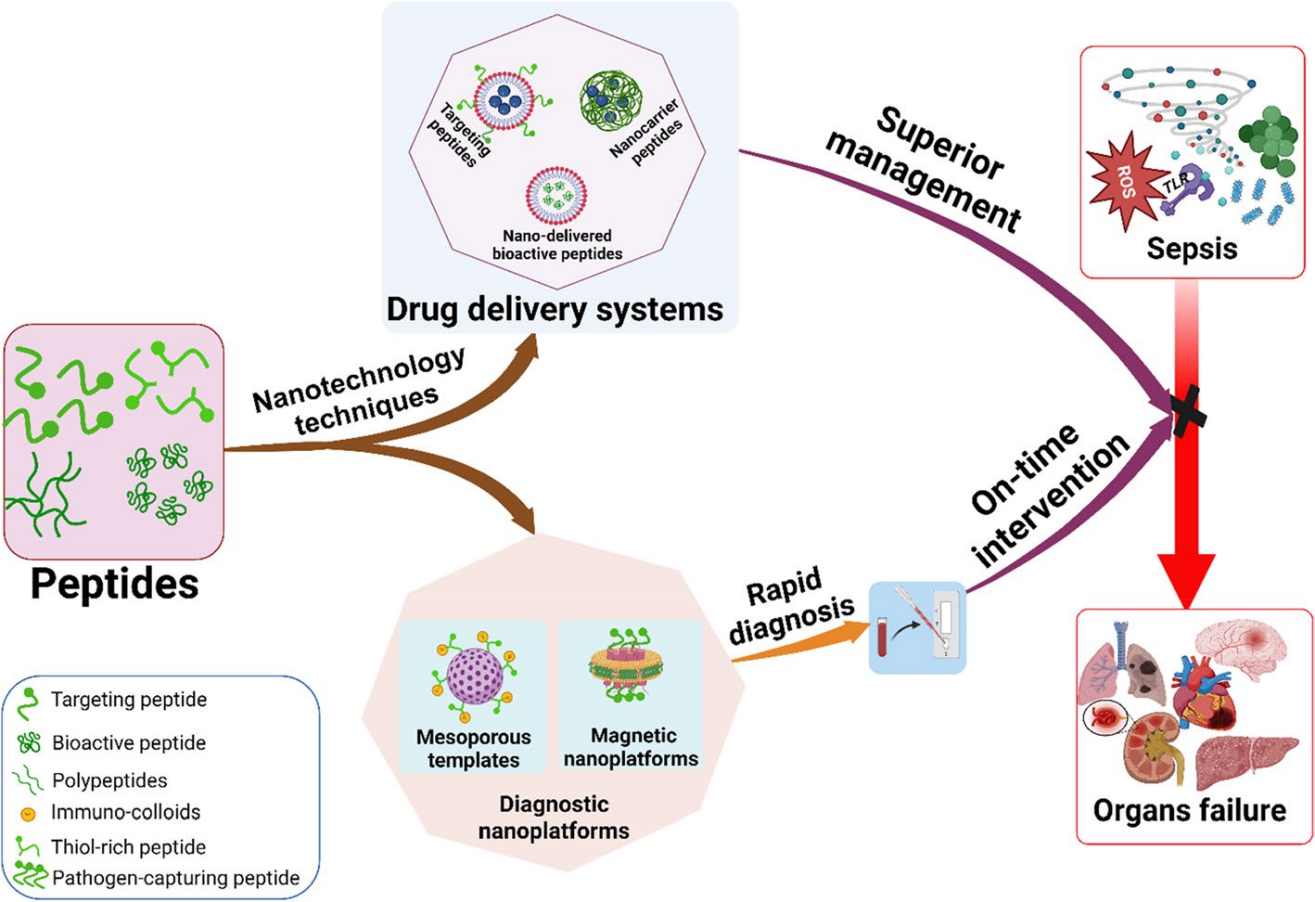

## Highlights

• Due to their unique characteristics, Peptides hold significant promise as part of nanotechnology approaches for diagnosing and treating sepsis, a current leading global killer.

• Various diagnostic nanotools utilizing peptides as pathogen recognition moieties can improve the pathogen capturing efficiency for sepsis diagnosis.

• Nano-delivery can overcome the limitations of bioactive peptides and enhance their antibacterial and anti-inflammatory efficacy in sepsis management.

• Peptides offer significant capabilities as targeting moieties and nanocarriers to augment the effectiveness of antisepsis agents.

• Future research identified can potentiate the applications of peptides for the diagnosis and treatment of sepsis.

## Introduction

Bacterial infections are still a major public health concern [1]. As per WHO reports, infections due to antimicrobial-resistant organisms resulted in 1.27 million deaths in the year 2019 and contributed to 4.95 deaths globally [2]. It is estimated that 700,000 people die each year worldwide, which is expected to rise to 10 million by 2050 [3]. The problem of mortality and morbidity of bacterial infections is made worse due to associated complications such as sepsis [4]. An estimated 48.9 million incident instances of sepsis were reported globally in 2017, leading to 11 million fatalities, accounting for 19.7% of all deaths worldwide [5]. With the emergence of the COVID-19 pandemic, antimicrobial resistance continues to gain ground and exacerbates bacterial sepsis, which is now the leading cause of death from infections [6]. If not detected and treated timely, sepsis can progress to septic shock, multiple organ failure, and death due to cardiovascular, coagulation, and endothelial dysfunction [7]. Accordingly, sepsis is a critical worldwide health problem with life-threatening implications, necessitating immediate attention to developing novel and powerful diagnostic and therapeutic strategies.

Nanoplatforms are providing new avenues for critical illnesses diagnosis and treatment [8]. These platforms offer cutting-edge approaches for disease diagnosis, enhancing sensitivity and decreasing processing time Without the necessity for specialized expertise [9]. Moreover, nanoplatforms can be fine-tuned to overcome conventional dosage forms' limitations by enhancing loaded drug pharmacokinetic and pharmacodynamic characteristics through disease site targeting, stimuli responsiveness, and mimicking disease pathophysiology [10, 11]. These positive attributes enable the use of lower drug concentrations, co-loading of different drugs in the nanosystems, having multi-responsive systems that respond to different disease environments, hence reducing systemic toxicity and improving therapeutic effectiveness [12]. These distinguishing characteristics of nanoscale drug formulations make them promising candidates for enhancing the efficiency of existing conventional antibiotics against multidrug-resistant bacteria [13]. Compared to conventional preparations, nano-antimicrobial formulations have demonstrated superior outcomes in managing sepsis [14, 15]. Therefore, nanotechnology-based systems provide efficient tools to decrease the burden of bacterial infections and sepsis.

The advancements and improvement of nanosystems' functionality require synthesizing bio-functional materials that can be employed in formulating them. Peptides are emerging as useful biomaterials for the formulation of nanosystems [16]. Peptides are a class of biological molecules composed of short chains of around 50 amino acids or less joined together by amide bonds [17, 18]. Due to the infinite possibilities of joining amino acids, peptides can serve as pathogens and biomarkers capturing motifs, bioactive agents, or as excipients in making diagnostic biosensors and drug delivery systems. As components in nanotools for sepsis diagnosis, peptides can be designed to have specific binding with high affinity to causative pathogens and sepsis-released inflammatory biomarkers, thus making diagnostic procedures more efficient and prompter [19]. Furthermore, peptides can be incorporated within these nanoplatforms to improve the stability and binding of sepsis biomarkers-capturing immune-colloids to mesoporous nano-templates for sepsis immunoassays [20]. Therefore, peptide-based nanoplatforms hold promising potential in advancing sepsis diagnosis, allowing for efficient and rapid interventions that will improve patient outcomes.

In sepsis management, bioactive peptides have been found to exhibit therapeutic and protective properties against sepsis and so provide effective new treatment options for patients suffering from this deadly condition [21, 22]. Among different classes of bioactive peptides, antimicrobial peptides (AMPs) are naturally existing peptides that have the ability to fight microbial infections and their related complications, such as sepsis [23]. Due to their novel antimicrobial modes of action, robust antimicrobial efficacy, minimal drug residual, and simplicity of production and modification, AMPs have held significant potential as a promising alternative to antibiotic therapy over decades [24]. More importantly, it is shown that antimicrobial resistance levels developed by AMPs are substantially lower as they target a variety of mechanisms that are not targeted by traditional antibiotics [25]. Additionally, anti-inflammatory peptides (AIPs) demonstrated beneficial effects in bacterial infections and sepsis management. The use of these peptides has been shown to help reduce inflammation by targeting various sites in the sepsis inflammatory cascade, thus reducing the amount of tissue and organ damage associated with sepsis and making the treatment more effective [26–30]. Overall, the unique properties and potential for applying AMPs and AIPs in bacterial infections and sepsis make them a promising area of research for developing new treatments. Nevertheless, bioactive peptides have certain drawbacks regarding bioavailability and tolerability (Teixeira et al. 2020), and research focuses on improving their potency and safety profile.

One of the ways the therapeutic profile of bioactive peptides is being improved is through encapsulation in nano-delivery systems [31]. Moreover, due to their diversity and ability to produce secondary nanostructures, bioactive peptides may be modified to form nanomaterials with enhanced characteristics [32, 33]. Apart from their biological activity and due to their superior physical, chemical, and biological characteristics, peptides have emerged as a potential constituent for the development of nanosystems for targeted delivery of drugs and genes [34–36]. Peptides can be employed in drug delivery technologies as nanocarriers, cell penetration enhancers, and targeting agents [17, 36]. Consequently, peptide-based nano-delivery systems have been developed and applied to treat a wide range of illnesses, such as sepsis, cancer, viral infections, and immune system disorders [37–42].

Numerous review articles have highlighted the application of peptides in nanotechnology-based bacterial infection management [43, 44] and the use of nanotechnology for AMPs delivery against general bacterial infections [45–49]. Additionally, several publications have reviewed the use of nanotechnology to manage sepsis [14, 19, 50, 51]. To the best of our knowledge, no review has discussed the various applications of peptides in nanosystems for diagnosing and managing bacterial sepsis.

Therefore, this review focuses on the numerous applications of peptides in nanosystems to identify and control bacterial sepsis. Initially, a theoretical background about the pathophysiology of sepsis, challenges associated with sepsis diagnosis and management, drug targets in sepsis, and peptides' physicochemical properties and their potential for application in nanotools against sepsis are presented. In addition, following a thorough search of many scientific databases, we discuss and critically analyze different applications of peptides in nanotechnology for sepsis diagnosis and management. The studies have been organized into two main sections, viz. diagnostic and management peptides-based nanosystems. We have further systematically categorized the nanosystems for sepsis management according to the role of peptides into: (i) nano-delivered bioactive peptides; (ii) peptides as targeting moieties on the surface of nanosystems; (iii) peptides as nanocarriers for antisepsis drug. Finally, this review highlights the challenges, gaps, and future perspectives to maximize the potential of applying peptides in nanotechnology tools to improve sepsis diagnosis and management.

## Background

A clear understanding of the pathophysiology of sepsis and potential drug targets is critical for developing new effective sepsis diagnostic and therapeutic tools. This section will discuss the pathophysiological background of sepsis, including the inflammatory pathways triggered by invading microorganisms and the consequences of that on the structure and function of body organs. The current trends in sepsis diagnosis and management and potential drug targets for sepsis management will also be discussed, accompanied by their challenges. Finally, the physicochemical and biological properties that make peptides a potential component of nanotools for sepsis diagnosis and management are covered.

### Pathophysiology of sepsis

**Fig. 1:**
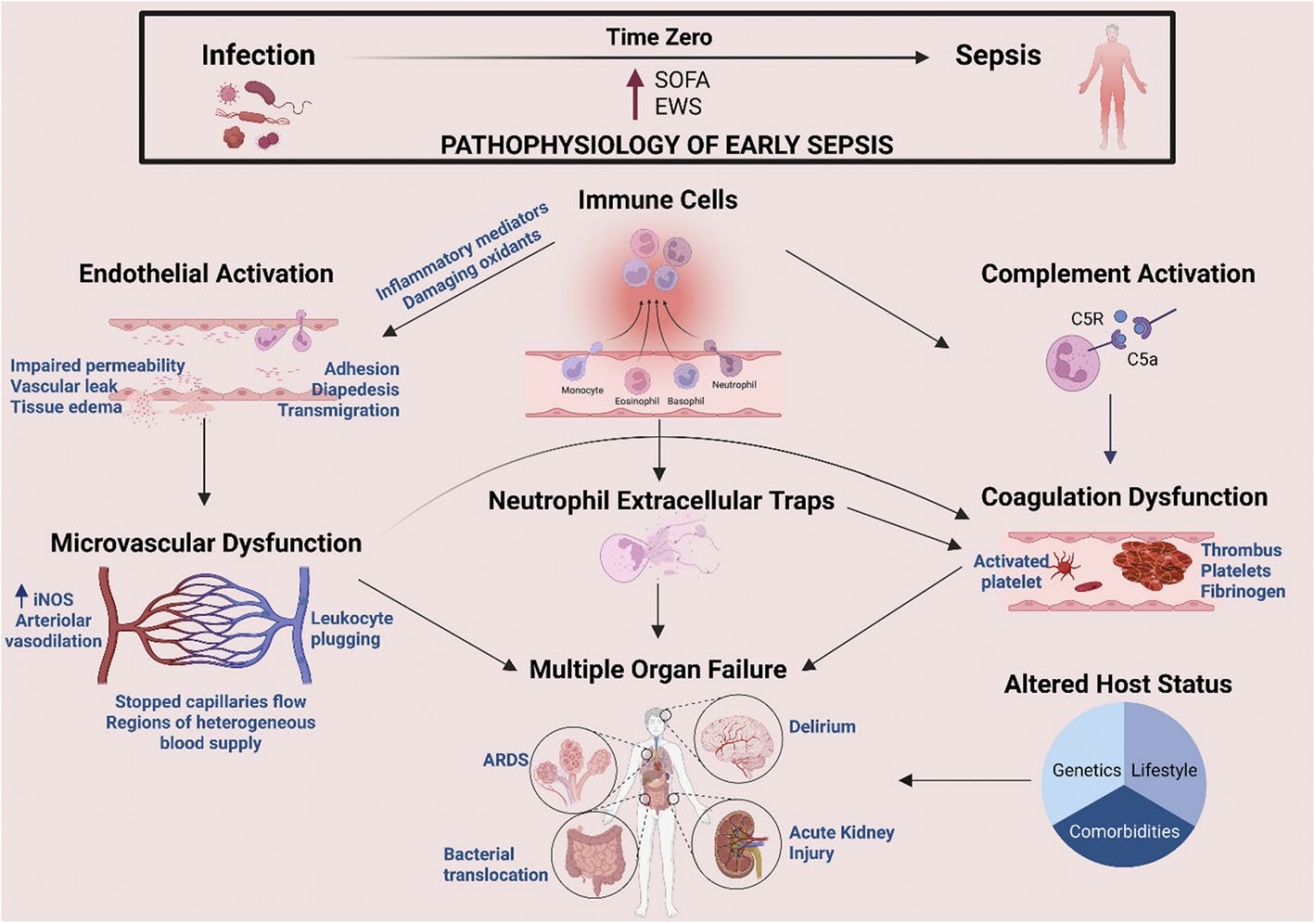
Immune responses in sepsis owing to infection. Illustration of converting to sepsis from infection. Immune cells activation results in the overproduction of inflammatory mediators that induce detrimental changes in cells and tissues, leading to multiorgan dysfunction and failure (SOFA: sequential organ failure assessment; EWS: early warning score; iNOS: inducible nitric oxide synthase; ARDS: acute respiratory distress syndrome) (Adopted with permission from [55]

Sepsis is a medical emergency and a life-threatening condition associated with a global disease burden [52]. Despite all experimental and clinical research efforts, sepsis remains one of the leading causes of morbidity and mortality in critically ill patients [53]. In the Third International Consensus (Sepsis-3), sepsis is defined as "organ dysfunction caused by a dysregulated host response to infection", highlighting for the first time the critical role of immune responses in the establishment of the illness [7]. After the invasion of microorganisms into the body, an immune response is triggered to fight off the invading microorganisms. This causes inflammation, a normal and necessary response to promptly identify, eradicate, and keep the infection localized [54]. However, as shown in Fig. 1, the immune response is exaggerated during sepsis, resulting in collateral damage and death of host cells and tissues, compromising both the affected and distant organs and leading to functional abnormalities and life-threatening multiorgan failure [55]. The pathophysiology of sepsis is generally defined as an early hyperinflammatory state that lasts many days, followed by a longer immunosuppressive state [56]. These two stages are connected with higher mortality, with the highest death rate in the early phase attributable to an enormous inflammatory response (cytokine storm) [14].

The over-released inflammatory mediators during the cytokine storms lead to significant damage to the endothelium and disruption of it is barrier function, vasodilation, activation of coagulation pathways, platelet aggregation and adhesion, and mitochondrial dysfunction [57, 58]. Overall, the dysregulated inflammatory-immune responses and their consequences mentioned above eventually lead to the formation of microvascular thrombi, hypotension, impaired cellular functions, local perfusion defects, tissue hypoxia, and progressive tissue damage, which finally cause refractory shock and multiorgan failure [59–62]. Cardiovascular Dysfunction, acute lung injury and acute respiratory distress syndrome, acute kidney injury, hepatic dysfunction, and CNS dysfunction and encephalopathy are well-known complications of sepsis, and their underlying mechanisms are reported in the literature [63–68]. These alterations and dysfunctions in the tissues and organs collectively contribute to much of the morbidity and mortality of sepsis [69].

Although the advancements in therapeutic approaches have enhanced the survival rate in the early phase of the exaggerated inflammatory response, current patterns in sepsis indicate that mortality arises during the subsequent stage of a compensatory immunosuppressive response when there is a shift toward an overall anti-inflammatory milieu [56, 70]. This post-sepsis immune paralysis involves various quantitative and functional defects of immune cells as a result of uncontrolled apoptosis of lymphocytes and decreased immunoglobulin production, which is linked to an increased susceptibility to secondary infections and organ injury and/or failure [14, 60, 71, 72]. Immunosuppression can last months after the septic event and is associated with increased mortality [73, 74]. The detailed sepsis pathophysiology and different involved pathways have been widely discussed in the literature, and readers are referred to them for more details [55, 67, 69, 73, 75–77].

### Sepsis diagnosis and management

Sepsis is considered a medical emergency that, if not diagnosed in its early stages, will result in a poor prognosis with increased morbidity and mortality [78]. The current diagnosis of sepsis relies on clinical evaluation, blood or urine cultures, and detection of inflammatory response biomarkers such as C-reactive protein (CRP), procalcitonin (PCT), and interleukin 6 (IL-6) [19]. However, the currently used biomarkers are not specific, and none have proven to be a specific sepsis indicator [79]. Moreover, the microbial cultures take a long time, and results may come out after 72 hours, making rapid sepsis diagnosis difficult [80]. Starting sepsis management as early as possible is critical to avoid complications and multiorgan failure [81]. As the current diagnostic tools for sepsis have such a delay, the empirical administration of intravenous broad-spectrum antibiotics is a usual initial intervention together with other additional therapies (e.g., anti-inflammatory (corticosteroids) and venous thromboembolism prophylactics) and measures for ventilation and hemodynamic stabilization (e.g., oxygen, albumin, and vasopressors administration and fluid resuscitation) [82]. However, the empirical use of broad-spectrum antibiotics with the uncertainty of diagnosis results and difficulties in differentiating infectious sepsis from noninfectious inflammations [83] will result in unwanted side effects for the already stressed patient and increased risk of antimicrobial resistance development [81, 84]. Putting all these challenges together raises the urgent need for new and specific sepsis diagnostics and management approaches.

### Drug targets in sepsis

As mentioned above, treating sepsis involves a combination of antibiotics to fight the underlying infection and supportive care to address the systemic inflammation and organ dysfunction that can occur because of the condition [85]. One of the key challenges in treating sepsis is identifying effective drug targets that can help reducing inflammation and tissue damage [86]. Besides targeting the invading microorganisms with antibiotics, several drug targets have been identified and studied in sepsis management [87, 88]. One of the main drug targets in sepsis is the inhibition of inflammatory mediators that play a critical role in developing sepsis, including cytokines, chemokines, and other inflammatory signaling molecules [89]. Drugs that target these inflammatory mediators have been developed and tested as potential treatments for sepsis [90]. For example, monoclonal antibodies that neutralize TNF-α, such as infliximab and etanercept, have been shown to improve outcomes in patients with sepsis [91]. Similarly, drugs that inhibit the activity of IL-1 (e.g., IL-1 receptor antagonist), IL-6, and IL-8 have also been shown to improve outcomes in patients with sepsis [92].

The coagulation cascade is another critical therapeutic target in sepsis [93]. As sepsis is associated with a hypercoagulable state, targeting the coagulation process with drugs such as anticoagulants or clotting factor inhibitors can prevent micro-clots formation and improve outcomes in sepsis [94]. Drugs that target the coagulation cascade, such as activated protein C (APC) and thrombin inhibitors, have been studied as sepsis therapies and demonstrated to enhance sepsis outcomes [88, 95]. Furthermore, the endothelial cells that line blood vessels play a crucial role in the body's reaction to inflammation and infection [96]. As a result, targeting the endothelium to manage sepsis is an active area of research [96]. In sepsis, the dysfunction of these cells can lead to increased permeability of blood vessels and decreased blood flow and so leakage of fluid and plasma protein into the tissues, resulting in hypotension and organ dysfunction [97]. One of the key pathways activated in the endothelium during sepsis is the nitric oxide (NO) pathway, leading to excessive vasodilation and decreased blood pressure [98]. Drugs that target the NO pathway, such as nitric oxide synthase inhibitors, have been investigated as potential treatments for sepsis [99]. Another promising strategy is the use of drugs that can improve endothelial function and reduce inflammation [100]. For example, endothelial protective agents, such as statins, have been shown to reduce inflammation and improve blood flow in sepsis [101]. In addition, new approaches, such as using extracellular vesicles as drug carriers for targeting the endothelium, are also being explored [102].

The complement system, a part of the immune system that helps identify and eliminate foreign invaders such as bacteria and viruses, is also one of the potential drug targets for sepsis management [103]. In sepsis, the complement system is overactivated, which can lead to inflammation and tissue damage [104]. As a result, targeting the complement system has been investigated for managing sepsis [105]. One approach to target the complement system in sepsis management is using complement inhibitors, which are drugs that block the activation of the complement system [106]. For example, eculizumab, a monoclonal antibody that targets the complement protein C5, has been shown to reduce the incidence of death and organ failure in patients with sepsis caused by meningococcal infections [107].

Bacterial toxins such as lipopolysaccharides (LPS) from Gram-negative bacteria, exotoxins from Gram-positive bacteria, and superantigens from both Gram-positive and Gram-negative bacteria are also potential targets in the management of sepsis [108]. Bacterial toxins can contribute to the development of sepsis by triggering the release of inflammatory cytokines and damaging vital organs and tissue, leading to septic shock and death [109]. Inhibiting the production or activity of these toxins can prevent toxicity and improve outcomes in sepsis management [108]. Various strategies are being studied, such as blocking toxins' binding to host cells, inhibiting their production, or neutralizing them using toxin-binding proteins or immunoglobulins [90, 110]. Even though developing therapies that target bacterial toxins is a promising area of research for treating sepsis, more research needs to be done to understand how these drugs work fully and if they are safe to use in clinical settings.

Overall, managing sepsis remains a complex and challenging task, and there is currently no single drug that can effectively address all the different pathways and processes involved in the condition. Further research is needed to identify additional drug targets and to develop more specific and effective medicines for sepsis.

### Potential of peptides for use in sepsis

Several drugs have failed in the treatment of sepsis. However, continued research and improved understanding of sepsis pathophysiology, including the complex interactions between inflammatory, coagulation, and fibrinolytic systems, has accelerated the development of novel treatments [88, 111]. Some of these drugs being researched and developed are peptides that hold great promise as therapeutic agents for treating sepsis [112–114]. As amino acids can be used in infinite arrangements in peptide synthesis, then peptides can be designed to have a wide range of unique physicochemical and biological properties. These unique properties include: 1) High specificity: Peptides may be designed to specifically target specific pharmacological targets, making their action highly selective with reduced off-target effects [115]; 2) Ability to target multiple pathways: Peptides can target multiple pathways in the sepsis cascade, which can help reduce the chances of antimicrobial resistance [19]; 3) Biodegradability: Inside the body, peptides are broken down into smaller biocompatible components, making them biodegradable with fewer side effects than traditional small molecule drugs [116]; 4) Ease of synthesis: Peptides can be synthesized using modern techniques in a laboratory setting, making it possible to produce cost-effectively large quantities of specific peptides for drug development [116]; 5) Permeability and bioavailability: some peptides can cross cell membranes, which increases their bioavailability and allows them to target intracellular targets in sepsis pathophysiology [117].

The properties mentioned above make peptide synthesis a promising approach for developing new diagnostic and management tools that can efficiently diagnose sepsis and effectively target both the bacteria causing sepsis and the pathophysiological pathways involved in the disease. For example, peptides have been designed to have strong and specific binding affinity to certain pathogens and inflammatory biomarkers, making them excellent capturing motifs for the diagnosis of sepsis [118]. Moreover, antimicrobial peptides have been shown to have potent bactericidal activity against Gram-negative and Gram-positive bacteria that are commonly associated with sepsis [119]. In addition to their antibacterial properties, peptides can target the pathophysiological pathways involved in sepsis, such as inflammation, oxidative stress, complement system, and coagulation [114, 120]. Furthermore, the excellent physicochemical and biological properties of peptides make them hold great potential as drug delivery systems construction materials for antisepsis drug delivery [121]. For example, peptides for drug delivery can be designed to respond to the characteristic microenvironment of sepsis, including acidity and high concentrations of reactive oxygen species (ROS) [121]. This allows for site-specific drug release, so good therapeutic outcomes with low side effects can be achieved with small doses of drugs that improve patient compliance and decrease the treatment cost. As a result, the application of peptides in sepsis diagnosis and management is an active area of research with promising outcomes that make them an attractive option in the battle against sepsis.

### Application of peptides in nanotools for sepsis diagnosis and management

**Fig. 2:**
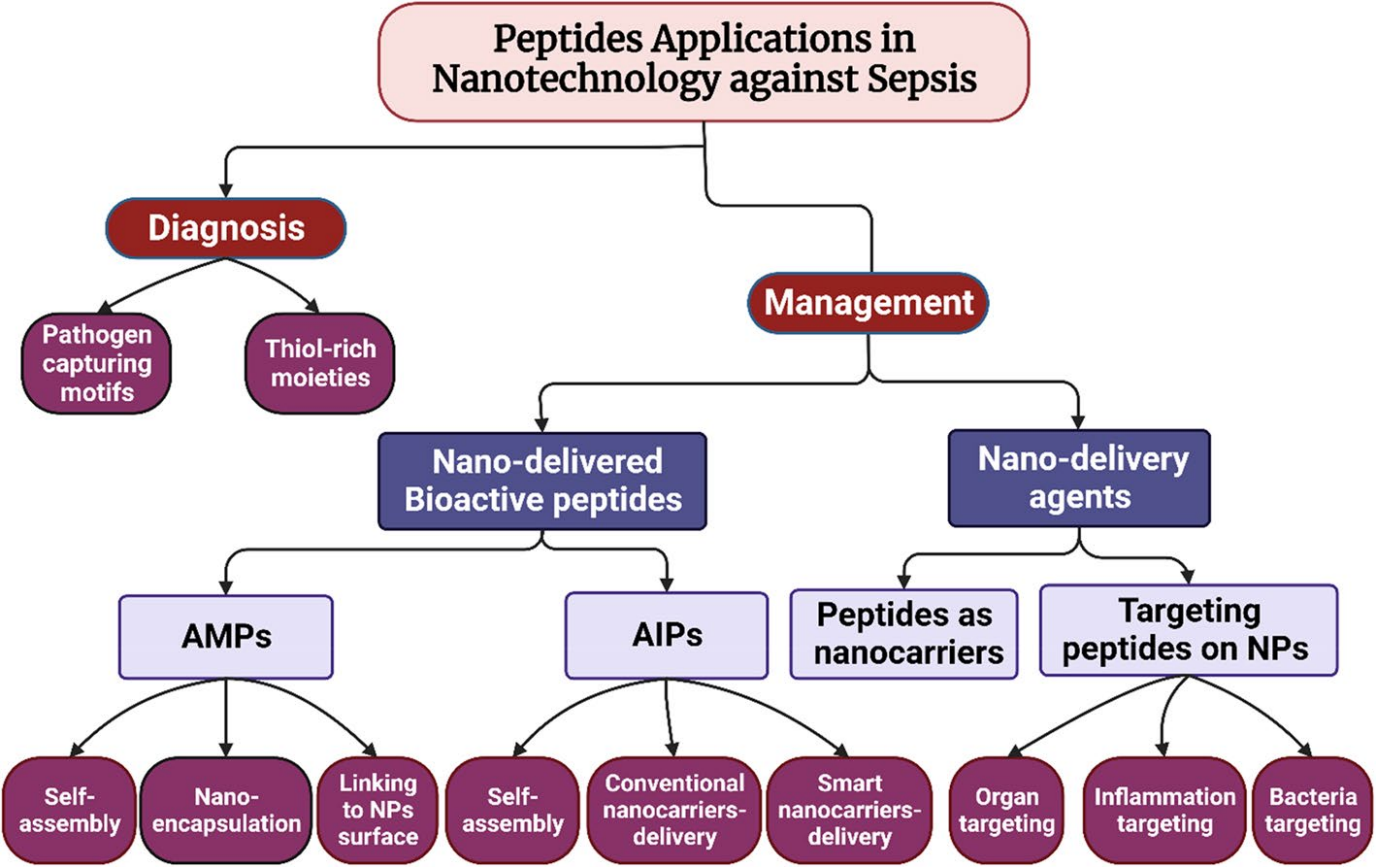
A schematic illustration of various applications of peptides in nanotools for sepsis diagnosis and management (NPs: Nanoparticles; AIPs: Anti-inflammatory Peptides; AMPs: Antimicrobial Peptides) (Created with BioRender.com)

Sepsis continues to pose a significant global health challenge despite remarkable advancements in medical technology. Its high mortality rates and limited effective diagnosis and treatment approaches underscore the urgency to develop new, efficient, and novel techniques for accurate diagnosis and timely intervention [79, 122]. The application of nanotechnology-based tools presents a multitude of opportunities for advancing sepsis diagnosis and management [14]. With their distinctive properties, peptides have emerged as promising candidates for developing nanotools to combat critical illnesses like sepsis [112, 114]. Figure 2 provides a visual representation of the multiple roles peptides play as components within nanosystems for sepsis diagnosis and management. This section aims to explore and critically review the diverse range of research studies that have employed peptides as integral elements of nanotools for sepsis diagnosis and management, encompassing both pharmacological and pharmaceutical applications.

### Peptides in nanotechnology for sepsis diagnosis

The timely diagnosis of sepsis is crucial to ensure effective treatment outcomes [78]. Although they can offer a promising avenue for early detection of sepsis, the use of peptides in developing nanotechnology tools for sepsis diagnosis is still in its infancy. Table 1 summarizes various studies done on the utilization of peptides in nanotools for sepsis diagnosis, highlighting the type of nanosystem, the peptide sequence, the role of peptide in the nanosystem, the targeted microorganisms or biomarkers, the mechanism of detection, the mode of investigation (in vitro and/or in vivo), and the key findings. As shown in the table, peptides have been mainly used for two different roles. The major role was using peptides as pathogen recognition moieties conjugated to the surface of magnetic or fluorescent nanoparticles to allow the capturing of bacteria and then separation or imaging. The other role has been the utilization of peptide as a thiol-rich moiety to enhance the binding of immuno-colloidal metallic nanoparticles to the surface of a mesoporous Surface-enhanced Raman scattering (SERS) template. It is clear that there is great potential for further research to uncover additional roles that peptides can play in nanotools for sepsis diagnosis. Moreover, most studies have focused on Gram-positive bacteria, indicating the need to address Gram-negative bacteria, which significantly influence the development of bacterial sepsis. The studies will be discussed according to the role of peptides in the nanosystem in the following subsections.

**Table 1:** Summary of peptides utilization in nano-diagnostics for bacterial sepsis

**System**	**Peptide**	**Role of peptide**	**Targets**	**Mechanism of detection**	**Proof of efficacy**	**Key findings**	**Reference**
Dendrimer-based magnetic nanoplatforms	Vancomycin (glycopeptide)	Pathogen capturing motif	*S. aureus* *L. monocytogenes*	Capturing of bacteria followed by Magnetic isolation and enrichment	In vitro	Rapid bacteria isolation in two minutes with more than 90% capturing efficiency for both bacteria. A limit of detection for *L. monocytogenes* and *S. aureus* of 32 and 41 CFUmL-1, respectively.	[123]
SPIONs	Pep1 (RKQGRVEVLYRASWGTV) Pep2 (RKQGRVEILYRGSWGTVC)	Pathogen capturing motif	*E. coli* *S. marcescens* *S. entérica* *S. enteritidis* *P. aeruginosa* *S. aureus*	Capturing of bacteria followed by Magnetic isolation and enrichment	In vitro	166 nm hydrodynamic diameter; peptide functionalization led to the formation of nanoparticle agglomerates of 1679 nm diameter. A remarkable depletion in cytokines (TNF-α, IL-6, IL-1β, Il-10, and IFN-γ) release. Capturing efficiency of over 60% for *S. aureus*, above 50% for *E. coli* and *S. marcescens*, and 35% for *P. aeruginosa*.	[118]
PEGylated magnetic nanoclusters	*S. aureus* binding peptide (VPHNPGLISLQG)	Pathogen capturing motif	*S. aureus*	Capturing of bacteria followed by Magnetic isolation and enrichment	In vitro	An average diameter of 150.8 ± 1.8 nm. Good cytocompatibility and magnetic properties. Capturing efficiency of more than 70% within 10 minutes	[124]
Fluorescent quantum dot (QD)	A modified auto-inducing peptide (AIPq)	Pathogen capturing motif	*S. aureus* *S. epidermidis* *S. saprophyticus S. haemolyticus*	Capturing of bacteria followed by imaging	In vitro (binding characteristics; biofilm imaging) In vivo (localization of MRSA in mouse model)	Higher selectivity towards accessory gene regulator (AGR)-positive virulent strains of *S. aureus* than the mutant strain. Effective penetration and imaging of biofilm-embedded colonies. Successful in vivo imaging of MRSA in infected mice.	[125]
Mesoporous SERS-substrate	Cysteine-rich peptide ((243bp) (GBS101000616.1))	To allow binding of immune-colloid gold nanoparticles to the surface of the mesoporous template.	Sepsis biomarkers	SERS	In vitro	A strong binding of gold nanoparticles to the surface of the mesoporous template. The SERS spectra exhibited the unique peaks of the three biomarkers. Low limits of detection for CRP (27 pM), PCT (103 pM), and sTREM1 (78 pM).	[20]

*SPIONs* Superparamagnetic iron oxide nanoparticles, *MRSA* Methicillin-resistant staphylococcus aureus, *SERS* Surface-enhanced Raman scattering

#### **Peptides as pathogen capturing motifs on nanoplatforms**   

To date, various approaches for detecting, capturing, and separating bacteria from blood have been developed to improve the diagnosis and management of bloodstream infections [126, 127]. Various pathogen recognition molecules, such as aptamers, antibodies, oligonucleotides, and carbohydrates, have been used to modify nanoplatforms for pathogens detection and separation [19, 128, 129]. Since the bacterial surface displays unique molecular compositions, a well-designed peptide can have a specific and robust interaction with an epitope or a receptor on the bacterial walls. When engineered on the surface of nanoparticles, these peptides will yield an efficient nanotool for pathogen capturing with improved efficacy in sepsis diagnosis [130]. The subsequent paragraphs will discuss the findings of various studies that have investigated the utilization of peptides as capturing motifs exhibited on magnetic nanoparticles or fluorescent quantum dots to fabricate nanoplatforms for pathogen detection and then isolation or imaging.

**Fig. 3:**
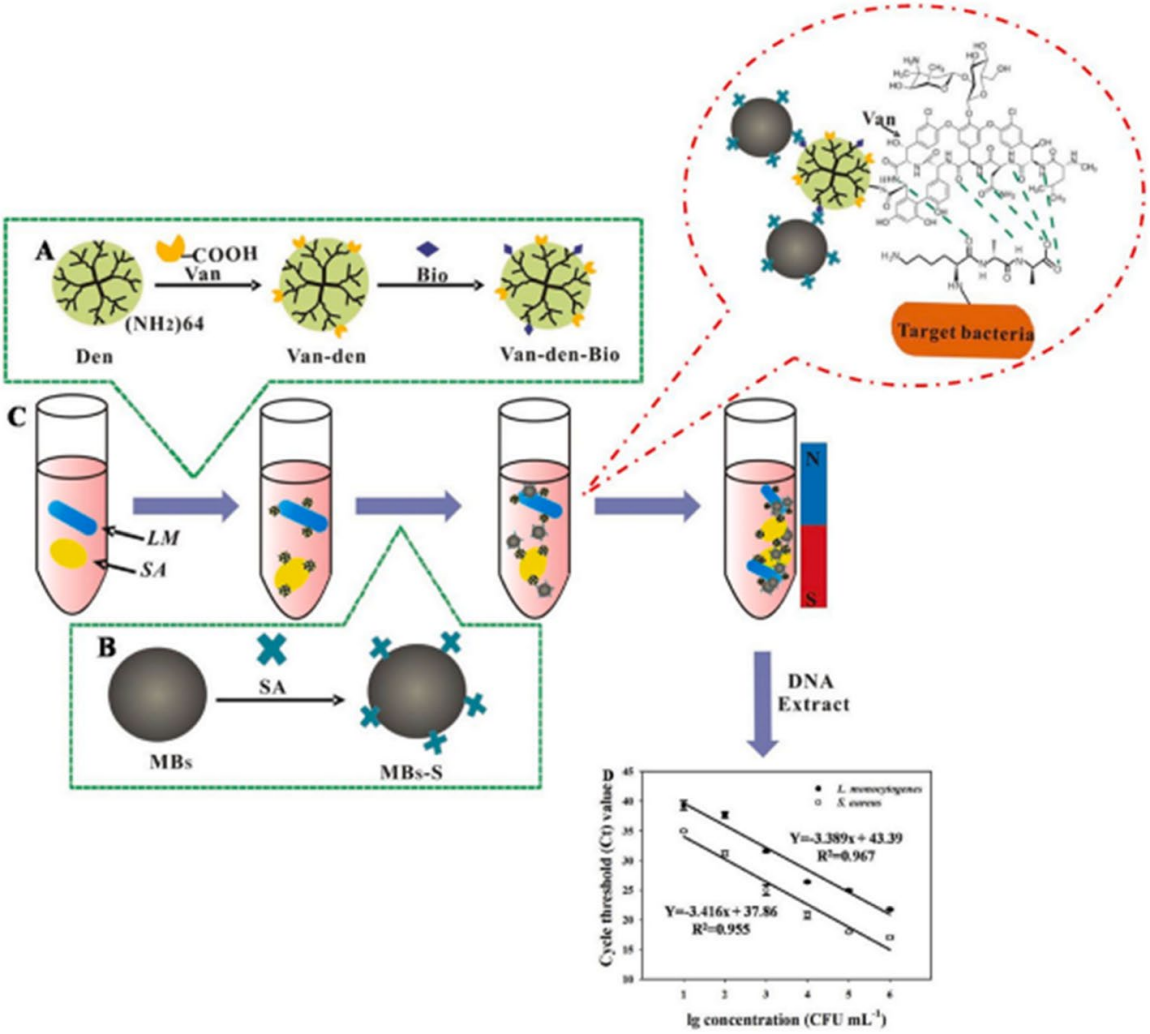
Detection of *S. aureus* and *L. monocytogenes* using a two-step approach paired with m-qPCR, utilizing the MBs-S~Bio-den-Van~bacteria complex [123]

Magnetic nanoparticles are promising nanoplatforms that can be effectively engineered with peptides as pathogen-capturing motifs for detecting and separating bacteria from human samples in simple and controllable processes [79, 131, 132]. Among different magnetic nanoparticles, magnetic beads (MBs), known for their superparamagnetic characteristics, have demonstrated their versatility in detecting, purifying, and analyzing analytes from intricate matrices. In order to achieve selectivity, MBs can be complexed with ligands such as peptides, aptamers, and antibodies to target the concerned pathogen selectively [133]. To this end, Feng et al., as illustrated in Fig. 3, have developed a vancomycin (Van)-modified magnetic nanoplatform composed of a dendrimer (G4 PAMAM) anchored with biotin and complexed with streptavidin-modified magnetic beads (MBs-S) to detect and isolate *L. monocytogenes* and *S. aureus* from human blood samples [123]. The glycopeptide antibiotic vancomycin (Van) can interact with the surface of the bacterial wall via hydrogen bonding and works as a pathogen recognition molecule [134].

They combined the MBs-S~Bio-den-Van platform with a multiplex quantitative PCR (m-qPCR) to enrich and identify the isolated pathogens. The platform demonstrated rapid bacteria isolation within 2 minutes and exhibited a capturing efficiency of 93.14% for *L. monocytogenes* and 94.58% for *S. aureus* from spiked healthy donors’ whole blood. The platform showed high sensitivity with limits of detection of 32 and 41 CFUmL^-1^ for *L. monocytogenes* and *S. aureus*, respectively. Their findings exhibited several potential advantages for bacterial detection in septic patients, including processing simplicity, low cost, high stability and specificity, and low detection limit. However, it should be noted that Van has the ability to bind to different types of Gram-positive bacteria, but it cannot bind to Gram-negative bacteria because of the differences in the components of their outer cell wall. Therefore, we believe this nanoplatform efficiency would be limited in cases of Gram-negative or mixed infections due to the specificity of Van to Gram-positives bacteria.

Another type of magnetic nanoparticles that can be modified with peptides to capture and separate bacteria from human samples are superparamagnetic iron oxide nanoparticles (SPIONs). SPOINs' non-toxicity, controllable size, large surface area-to-volume ratio, and ability to be functionalized with targeting moieties make them promising tools for early detection and diagnosis of diseases [135, 136]. For instance, Friedrich and coworkers developed a 3-aminopropyl triethoxysilane (APTES)-coated superparamagnetic iron oxide nanoparticles modified with bacterial cell wall-binding peptides for bloodstream bacterial pathogens separation [118]. The two peptide sequences (Pep1 (RKQGRVEVLYRASWGTV) and Pep2 (RKQGRVEILYRGSWGTVC)) were obtained from the salivary glycoprotein GP-340, which is known to interact with bacterial cell wall components [137]. A modified one-step coprecipitation approach was used to produce the SPION-APTES with a hydrodynamic diameter of 166 nm, which formed nanoparticle agglomerates of 1679 nm diameter after peptide functionalization.

**Fig. 4:**
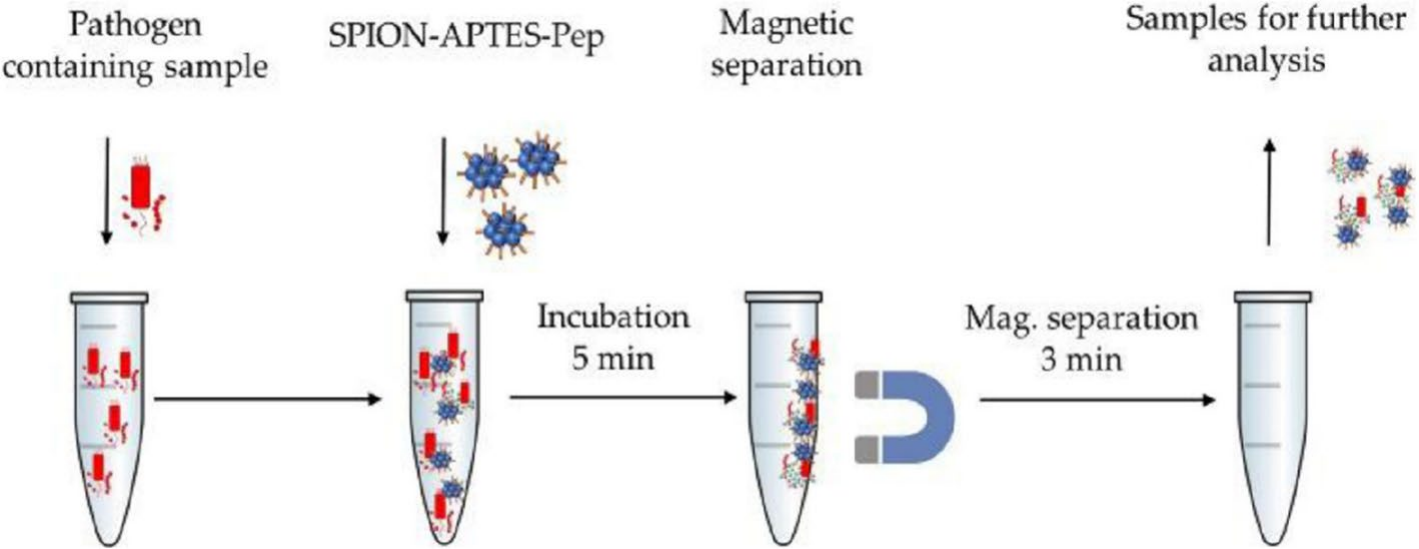
Experimental setup of separation of bacteria from blood using SPION-APTES-Pep (Adopted from [118])

The nanoparticles were found to be cyto- and hemo-compatible. As shown in Fig. 4, whole blood samples from healthy volunteers spiked with Gram-negative (*E. coli*, *S. marcescens, S. enterica, S. enteritidis*, and *P. aeruginosa*) and Gram-positive (*S. aureus*) bacteria were used to test the separation efficiency. *S. aureus* had an above 60% removal rate, *E. coli* and *S. marcescens* over 50%, and *P. aeruginosa* separation was only 35%. Besides the diagnostic role, the system showed a strong inhibition of cytokines (TNF-α, IL-6, IL-1β, Il-10, and IFN-γ) release. It is worth noting that this dual and simple theranostic approach could hasten both diagnosis and management for patients suspected to have sepsis. However, as the platform showed reduced efficiency in Gram-negative bacteria, we are of the view that this will limit its applicability, especially in the cases of mixed infections. Moreover, the separation efficiency was found to be affected by the anticoagulant employed and the concentration of Ca^2+^ ions in the blood collection tubes. As a result, it is imperative to underscore that this may affect the system's applicability in emergency situations where blood collection tubes with the suitable anticoagulant are unavailable.

To further improve the performance of magnetic iron oxide nanoparticles, coating with a layer of polyethylene glycol (PEG) can be performed. PEGylation provides improved stability, biocompatibility, and magnetic properties. PEGylation also reduces non-specific binding and potential immunogenicity, making the magnetic nanoparticles more suitable for various biomedical applications such as diagnosis and targeted drug delivery [138]. On this point, Pan et al. developed a peptide-modified PEGylated-iron oxide composite nanoclusters (peptide@PEG@MNCs) to isolate and identify *S. aureus* form blood samples [124]. The peptide (SA5-1: VPHNPGLISLQG) was chosen from a bacteriophage display library for its ability to selectively attach to the cell surface of *S. aureus*. SA5-1 was chemically bonded to the PEGylated magnetic nanoclusters (PEG@MNCs), resulting in the formation of the peptide@PEG@MNCs particles with an average diameter of 150.8 ± 1.8 nm and good cytocompatibility and magnetic properties. Human serum spiked with different bacterial strains, including *E. coli*, *P. aeruginosa*, *S. aureus* (susceptible and resistant strain), and *S. epidermidis,* was utilized to demonstrate the capturing efficacy under conditions simulating sepsis. A capture efficiency of over 70% was achieved for all tested microorganisms within 10 minutes. When a rinsing step was introduced to the process, only *S. aureus* was detected, notably, indicating the system's ability to selectively capture *S. aureus*, which was anticipated to the strong affinity of the peptide to *S. aureus* pathogens. Nevertheless, as the capture efficiency declined with increasing the bacterial concentration, we believe this may affect the applicability of the platform if high bacterial load exists in patients' blood samples.

In summary, the findings from the three studies indicate that peptides-modified magnetic nanoparticles have significant promise as a rapid and effective diagnostic tool for bacterial sepsis. Regarding efficacy, Feng et al.'s system showed a significantly higher capturing efficiency of *S. aureus* compared to the other two systems by Friedrich et al. and Pan et al. Although the second study by Friedrich et al. included Gram-negative bacteria in their evaluations, their system’s capturing efficacy was again higher for Gram-positive than Gram-negative bacteria. Interestingly, Pan et al. in their study were successfully captured both Gram-positive and Gram-negative bacteria and, at the same time able to enhance the system’s specificity toward Gram-positive bacteria by introducing a rinsing step; consequently, we contend that their approach was more flexible and comprehensive for sepsis diagnosis applications. Notably, Friedrich et al.'s system stood out for its unique ability to inhibit the release of cytokines, which will add value to the therapy of septic patients, in addition to its diagnostic use. Overall, the advantages and limitations of each one of them should be considered when applying them in clinical practice.

While the other three studies have used peptides as capturing motifs expressed on magnetic nanoparticles for bacterial separation from blood samples, another study conducted by Shrivastava and coworkers [125] applied the pathogen-capturing properties of peptides for in vivo imaging of bacteria inside the body. Imaging techniques for bacteria play an essential role in various aspects of diagnosing infections and understanding their pathogenesis [139]. Using in vivo imaging techniques, researchers can gain insights into the spatial distribution of bacteria within the host, track their movement, and understand the dynamics of infections [140]. Various molecular probes have been employed to target biological processes during infections. Nonetheless, visualizing bacteria in vivo remains challenging due to the inherent difficulty of targeting the specified bacteria directly [141]. Hence, there exists a necessity to identify highly efficient materials capable of precisely targeting specific pathways within bacterial internal processes that play a crucial role in their pathogenicity and so allow for efficient in vivo imaging of bacterial pathogens.

A potential solution to enhance the in vivo imaging of bacteria lies in using biomaterials capable of selectively targeting the quorum sensing (QS) communication employed by many bacteria to coordinate the expression of virulence genes during infections [140]. Based on that, Shrivastava and colleagues have developed a new fluorescent quorum-based nano-bio probe (QNBP) to monitor the localization of multiple-drug resistant *S. aureus* (MRSA) bacteria in vivo. An auto-inducing peptide (AIPq) with the ability to target the MRSA accessory gene regulator (AGR) QS system has been conjugated onto a fluorescent quantum dot (QD) surface to develop the QNBP system [125]. The nano-bio probe was assessed in vitro for bacterial binding characteristics and in vivo for imaging the bacteria in a mouse model. The QNBPs showed higher selectivity in binding to AGR-positive virulent strains than the mutant strain, thus confirming its suitability for in vivo imaging of pathogenic *S. aureus.* The QNBP was also capable of penetrating MRSA biofilm and effectively image embedded colonies, giving a new approach for identifying MRSA embedded in biofilms [142]. When employed as a fluorescence probe for in vivo imaging of MRSA in a systemic infection mouse model, QNBP resulted in the detection of robust fluorescent signals in most infected organs and high-quality fluorescence images were acquired post-infection. It is to be noted that this peptide-based nano-bio probe can offer new perspectives into exploration of infection pathways in vivo and aid in the diagnosis and management of life-threatening infections such as MRSA-induced sepsis. However, we argue that the need for advanced instruments such as fluorescence spectrophotometer and confocal laser scanning microscopy for recording the results may restrict the practical application of this system.

#### Peptides as thiol-rich moiety for binding of metallic colloids to mesoporous templates

Surface-enhanced Raman spectroscopy (SERS) is a surface-sensitive technique that enhances Raman signals of molecules adsorbed on metallic nanostructures such as plasmonic-magnetic silica templates. SERS templates modified with SERS- tags specific to certain disease biomarkers can effectively detect and analyze these biomarkers, aiding in disease diagnosis [143, 144]. Metallic colloids, such as gold nanoparticles, when functionalized with Raman dyes and disease biomarkers-specific antibodies and subsequently immobilized on plasmonic mesoporous templates, hold great promise as SERS-based diagnostic tools for the detection of biomarkers levels [145–147]. Bacteriophages have the potential for synthesizing mesoporous templates; however, to incorporate metallic colloids into them, their surfaces must be chemically modified with thiol donors, which affects their critical properties, such as assembly and binding [148]. To overcome this, thiol moieties, such as cysteine-rich peptides, can be displayed on the phage during synthesis without chemical modification [149]. In this regard, Nguyen and colleagues have developed a mesoporous SERS substrate based on M13KE phage displaying a cysteine-rich peptide (243bp: GBS101000616.1) as a template for sepsis biomarkers assay in human serum sample [20].

**Fig. 5:**
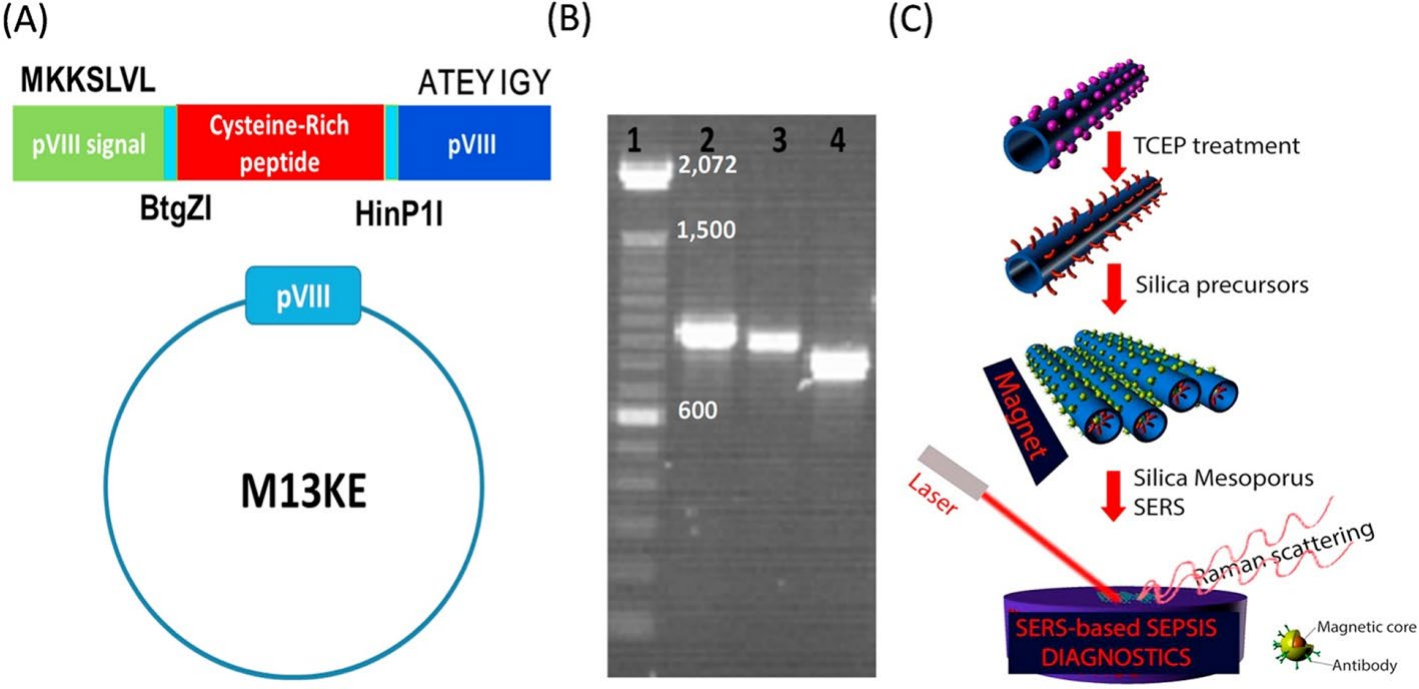
Representation of the process from phage display to manufacturing of the SERS substrate. **A** Insertion of the cysteine-rich peptide into the major PV111 protein domain of the M13KE phage using two restriction enzymes (BtgZI and HinP1I). **B** The colony PCR analysis on a 1% agarose gel confirms the effective integration of the cysteine-rich peptide into the pVIII region of the M13KE plasmid. **C** Utilization of cysteine-rich peptide phage display for the production of SERS substrates. The phage was decorated with an immuno-colloid made of gold-coated magnetic nano-stars (Au-MNS) after treatment with tris (2-carboxyethyl) phosphine hydrochloride solution (TCEP) to activate the thiol groups. The phage was polymerized with silica precursor to give amorphous biomaterial gel and then calcinated to form the mesoporous template. After incubation of the template with a serum sample spiked with sepsis biomarkers, the complexes were separated using a magnet and subjected to Surface-enhanced Raman scattering (SERS) measurement (adopted from [20])

As shown in Fig. 5, the surface of the template was magnetized with gold-coated magnetic nano-stars (Au-MNS) modified with a SERS-tag consisting of specific antibodies for three sepsis biomarkers (soluble Triggering Receptor Expressed on Myeloid cells-1 (sTREM1)), C-reactive protein (CRP), and procalcitonin (PCT) and a RAMAN dye for RAMAN signal amplification. The cysteine-rich peptide allowed strong binding of the Au-MNS when its thiol groups were reduced to active thiols. The SERS-based immunoassay was done on human serum samples, and the SERS spectra of the magnetically separated template exhibited characteristic peaks of the tags corresponding to the three biomarkers. The system demonstrated high sensitivity, excellent specificity, and low detection limits for CRP (27 pM), PCT (103 pM), and sTREM1 (78 pM). We believe this approach offers a potential alternate tool for the initial stages of sepsis monitoring. Despite this, it is to be mentioned that the fabrication complexity and risk of surface contaminants interference due to the high surface area of mesoporous templates could limit the scalability and reproducibility and interfere with the accuracy and reliability of SERS measurements in clinical settings.

To conclude, the studies discussed in this section demonstrated the crucial role of peptides in sepsis diagnosis by either offering pathogen recognition capacities or enabling the binding of metallic nanoparticles to mesoporous templates. These approaches offer several advantages, including enhanced selectivity and sensitivity in detecting sepsis-causing pathogens, as well as the ability to provide rapid results. Nevertheless, relying on blood samples from healthy volunteers to assess the developed nanoplatforms may not accurately reflect the complexity of sepsis cases. Therefore, it is imperative to validate the clinical applicability of the developed nanoplatforms by testing them on blood samples obtained from sepsis patients. Such validation would ensure these nanoplatforms' accuracy, reliability, and effectiveness in real-world clinical settings.

### Peptides in nanotechnology for sepsis management

Although numerous potential therapeutics for sepsis have been identified, achieving effective treatment remains a formidable challenge, and sepsis continues to threaten healthcare systems worldwide with its high mortality rates and complex pathophysiology [150]. In recent years, using peptides in nanotechnology has ignited new hopes for developing targeted and efficient therapeutic strategies for complex illnesses such as sepsis [151]. This section will delve into the developments and transformative potential of peptides in nanosystems to manage sepsis. The multifaceted applications of these peptides will be explored and discussed, including their utilization as bioactive agents with antimicrobial or anti-inflammatory properties delivered through nanosystems. Additionally, their role as nanosystem components, including nanocarriers for antisepsis drug delivery or as surface modifiers for nanosystems to target sepsis microenvironment or causative bacteria, will be highlighted and critically analyzed in the following subsections. An emphasis will be put on the manufacturing processes of nanosystems, key characterization and evaluation, and key findings of the studies.

#### Nano-delivered bioactive peptides

##### Antimicrobial peptides nanosystems

Antimicrobial peptides (AMPs) are short chains of amino acids, typically ranging from 5 to 50 residues, which may be regarded as natural antibiotics synthesized by various organisms, including mammals, plants, protozoa, fungi, and bacteria. They may have amphipathic or cationic structural composition and can display a wide range of antimicrobial activity, targeting both Gram-positive and Gram-negative bacteria, fungi, viruses, and protozoa [152]. A complete understanding of the mechanism of action of AMPs remains elusive. Nevertheless, many mechanisms have been proposed, suggesting diverse interactions with phospholipid membranes of microorganisms [153, 154]. In recent years, exploring AMPs as potential therapeutics has gained substantial momentum, driven by their remarkable antimicrobial activity and ability to overcome antimicrobial resistance [155]. However, the clinical translation of AMPs into effective sepsis treatments has been hindered by various challenges, including toxicity, limited stability and enzymatic degradation, and ununderstood pharmacokinetic profiles [156]. Therefore, robust delivery strategies are needed to allow effective use of AMPs in clinical settings.

The utilization of nanotechnology-based delivery systems has emerged as a promising trajectory for mitigating the aforementioned challenges and augmenting the therapeutic efficacy of AMPs. Literature has documented that nano-scaled delivered peptides exhibit diminished cytotoxicity, improved physiological stability, and increased efficiency at the desired target [157]. Hence, nano-delivery systems could enhance the physicochemical and pharmacological characteristics of AMPs, enabling their use as effective sepsis therapeutics in clinical practice. Table 2 illustrates different studies reported on nano-delivered AMPs for sepsis management, highlighting the type of nanosystem, the peptide sequence, the nano-delivery strategy, the targeted bacteria, the key evaluations, and the key findings. As illustrated, peptides have been either conjugated with other moieties to aid nanoscale self-assembly, encapsulated into nanosystems, or linked to the surface of nanoparticles. Evidently, most of the studies have focused on the self-assembly and nano-encapsulation of AMPs, indicating room for more research on the conjugation of AMPs to the surfaces of metallic and organic nanoparticles. The studies will be discussed in the following subsections according to the strategy of nano-delivery of AMPs.

**Table 2:** Summary of nano-delivered AMPs for bacterial sepsis management

**Nanosystem**	**Peptide**	**strategy**	**Targeted bacteria**	**Key evaluations**	**Key findings**	**Reference**
Self-assembled Nanobiotic	HD5	Self-assembly	*S. aureus* MRSA *E. coli* *K. pneumoniae P. aeruginosa* *A. baumannii*	DLS TEM Hemolysis assay. Cytotoxicity assay. In vitro and In vivo efficacy.	Shape: spherical; size: 56.0±8.4 nm. Improved hemocompatibility and biosafety profiles. Enhanced bactericidal activity. Rescued mice from sepsis by lowering the bacterial burden and alleviating organ damage.	[158]
Self-assembling chimeric peptide nanoparticles	PFPFPFP-KPKPKPKPKPKP-NH2	Self-assembly	*S. aureus* *E. coli*	DLS TEM Hemolysis assay. Cytotoxicity assay. In vivo biocompatibility. In vitro and In vivo efficacy.	Shape: spherical; size: 20-50 nm. Improved hemocompatibility and biosafety profiles. Lower MIC (*E. coli:* 7.3 to 12.3 μM; *S. aureus:* 5.3 to 10 μM). Superior reduction of bacterial load and pro-inflammatory cytokines level (TNF-α, IL-6, and IL-1β) in septic mice.	[159]
AMPNP	KR-12 (KRIVKRIKKWLR)	Co-assembly	*E. coli* *S. aureus* MRSA	DLS TEM In vitro and In vivo efficacy.	Shape: spherical; size: 80.82 ± 0.51 nm; ZP: 28.07 ± 2.25 mV. Broad spectrum in vitro antibacterial activity. Improved targeting of inflamed cells. Superior reduction in cytokine levels (IL-1β, TNF-α, and IL-6) and leukocyte infiltration into organs of septic mice.	[22]
AMPNP	KR-12 (KRIVKRIKKWLR)	Co-assembly	*S. aureus* MRSA *E. coli*	In vitro and in vivo bacterial targeting. In vitro and In vivo efficacy.	Improved Adhesion to and targeting of bacteria in vitro and in vivo. Broad spectrum antibacterial activity (90% bacterial killing). Improved survival rates of mice and significantly reduced cytokine levels (IL-1β, TNF-α, and IL-6) and leukocytes tissue infiltration.	[160]
Polymeric nanoparticles	Clavanin A (VFQFLGKIIHHVGNFVHGFSHVF-NH2)	Nano-encapsulation	*S. aureus* *K. pneumoniae P. aeruginosa*	DLS EE% Invitro antibacterial assays. In vivo efficacy against polymicrobial C57BL6 mice sepsis model (sub-lethal and Lethal doses)	Size:372 nm; ZP: -7.16 mV; PDI: 0.123; EE%: 98%. In vitro inhibition of bacterial growth: *S. aureus* (91%), *K. pneumoniae (*20%), P. aeruginosa (39.8%), *E. coli* (no effect). MIC (MRSA): 64 µg⋅mL^-1^. 100% mice survival rates for sub-lethal sepsis assays and 40% for lethal sepsis assays.	[161]
Polymeric nano-construct	Mastoparan (INLKALAALAKKIL-NH2)	Nano-encapsulation	*A. baumannii*	DLS EE% & LC In vitro and In vivo efficacy.	Size: 156 nm; ZP: +54.9 mV; EE%: 90.54%; LC: 22.63%. MIC: 4 μg/mL Improved physical activity of mice and reduced blood bacterial counts.	[162]
VLNPs	mRNA of AMP-IB367 (RGGLCYCRGRFCVCVGR_CONH2_)	Nano-encapsulation	Multi-drug resistant *S. aureus*	DLS EE% In vitro and In vivo efficacy.	Size: ≈ 140 nm; PDI: ≈ 0.1; ZP: ≈ 22 mV; EE%: ≈ 90%. In vitro bacterial growth inhibition of 87%. Significant reduction in blood bacterial load and improvement of survival rates of septic mice.	[163]
Gold nanoparticles	Cecropin melittin-cysteine (CM-SH: KWKLFKKIGAVLKVLC)	Linking to the surface of NPs	*S. aureus* *E. coli*	DLS Antimicrobial resistance development assay. In vitro and In vivo efficacy.	Size: 14 nm; PDI: 0.1 ± 0.02; ZP: 28 ± 2 mV. No spontaneous resistance development after 28 days of exposure of *E. coli* to sub-MIC. 4-fold reduction in MIC Significant reduction in bloodstream bacterial count and IL-10 levels.	[164]
Liposomes	Ts: GSKKPVPIIYCNRRSGKCQRM	Linking to the surface of NPs	*K. pneumoniae*	DLS In vitro and In vivo efficacy.	Size: 152.5 ± 3.2; PDI: 0.254; ZP: +5.3; EE%: 76.8 ± 2.7%. 2 to 8-fold reduction of MIC. Significant reduction in mice lethality rates after Ts-linking from 73.3% to 6.7%.	[165]

*HD5* Human alpha-defensin-5 peptide, *MRSA* Methicillin-resistant staphylococcus aureus, *DLS* Dynamic light scattering, *TEM* Transmission electron microscopy, *MIC* Minimum inhibitory concentration, *AMPNP* Antibacterial peptide polymeric nanoparticles, *TNF-α* Tumer necrosis factor-α, *IL-6* Interleukin-6, *IL-1β* Interleukin-1β, *ZP* Zeta potential, *CLP* Cecal ligation and puncture, *EE%* Encapsulation efficiency, *PDI* Polydispersity index, *MIC* Minimum inhibitory concentration, *LC* Loading capacity, *VLNPs* Vitamin C lipid nanoparticles, *NPs* Nanoparticles, *AMP-IB367* Antimicrobial peptide-IB367, *IL-10* Interleukin-10, *Ts* S-thanatin

##### Self-assembled AMP nanosystems

Designing peptides to self-assemble into nanostructures is a powerful strategy to bolster their stability, biosafety, and therapeutic outcomes, allowing their effective application in disease management [166]. The self-assembly of peptides is usually achieved by selecting amino acid series encompassing both hydrophilic and hydrophobic amino acids and linking them to yield amphiphilic structures capable of spontaneous self-assembly upon exposure to water. Alternatively, amphiphilic peptides can be engineered by conjugating them with hydrophobic motifs such as alkyl chains and fatty acids [166].

AMPs are among the bioactive peptides that can be designed to self-assemble and become more stable and effective [167]. In this regard, Lei et al. have successfully prepared self-assembled nanoparticles of the host-defense antimicrobial peptide, human alpha-defensin 5 (HD5), with improved stability, biosafety, and antibacterial effectiveness [158]. To introduce hydrophobicity and promote the nano-assembly of HD5, they conjugated myristic acid, a 14-carbon chain saturated fatty acid, to the C-terminus of HD5 to produce myristoylated HD5 (HD5-myr). HD5-myr spontaneously self-assembled in aqueous media, producing spherical-shaped nanoparticles -termed Nanobiotic- that were found to be hemocompatible and unlike the native HD5 resistant to proteolytic enzymes. The Nanobiotic exhibited significantly enhanced broad-spectrum bactericidal activity in vitro against different Gram-positive and Gram-negative bacterial strains, including *S. aureus,* MRSA*, E. coli, A. baumannii, P. aeruginosa, and K. pneumoniae,* compared to the free HD5*.* The Nanobiotic was stable and retained the antibacterial efficacy even in the presence of proteolytic enzymes and high salt concentration, while the free HD5 underwent extensive hydrolysis within 24 hours. In the in vivo studies, the Nanobiotic exhibited protective effects, effectively rescued mice from *E. coli*-induced sepsis, and improved their survival rates by reducing the overall bacterial load in the body and preventing organ damage. Their outcomes have shown that the supramolecular assembly of AMP to make nanoparticles has tackled peptides' instability problem and achieved good antibacterial activity both in vitro and in vivo. We believe this work provides a promising candidate for treating bacterial sepsis that can be simply scaled up and manufactured.

**Fig. 6:**
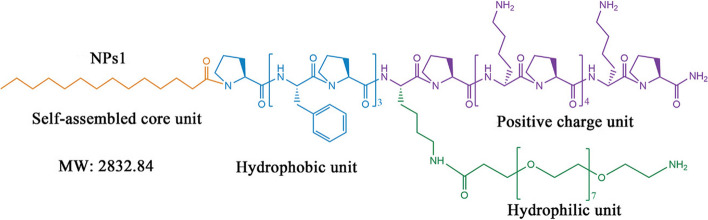
The structural design of self-assembling chimeric peptide. The peptide sequence comprises hydrophobic and cationic amino acids. The peptide is linked to the hydrophobic alkyl chain to enhance the self-assembly and the hydrophilic PEG unit to provide a stealth effect and improve biocompatibility (adopted from [159])

Utilizing the same concept of peptide-conjugates assembly into nanoparticles, Tan and coworkers designed self-assembling chimeric peptide nanoparticles to treat bacterial infection and sepsis [159]. As shown in Fig. 6, the AMP peptide sequence (PFPFPFP-KPKPKPKPKPKP-NH2) was linked to a 14-carbon alkyl chain to provide hydrophobic properties and modified with PEG domain at various locations to offer stealth effect and biocompatibility. The peptide amphiphiles self-assembled into nanoparticles of around 20-50 nm in diameter, which have shown good in vitro and in vivo biocompatibility. The self-assembled nanoparticles demonstrated broad-spectrum antibacterial activity against various strains of *E. coli* (MICs: 7.3 to 12.3 μM) and *S. aureus* (MICs: 5.3 to 10 μM) even in the presence of high concentrations of proteases and different salt conditions. Moreover, no spontaneous antimicrobial resistance to the peptide nanoparticles was detected when the *E. coli* ATCC25922 strain was subjected to sub-MIC dose treatment. In vivo, the nanoparticles have also demonstrated the ability to alleviate *E. coli* sepsis in mice and piglets and significantly reduced organs' bacterial load and the concentrations of pro-inflammatory cytokines (TNF-α, IL-6, and IL-1β). We argue that this approach provides an effective strategy to accelerate the clinical translation of newly developed peptides to meet the real need for effective sepsis therapies and to fight the growing antimicrobial resistance.

**Fig. 7:**
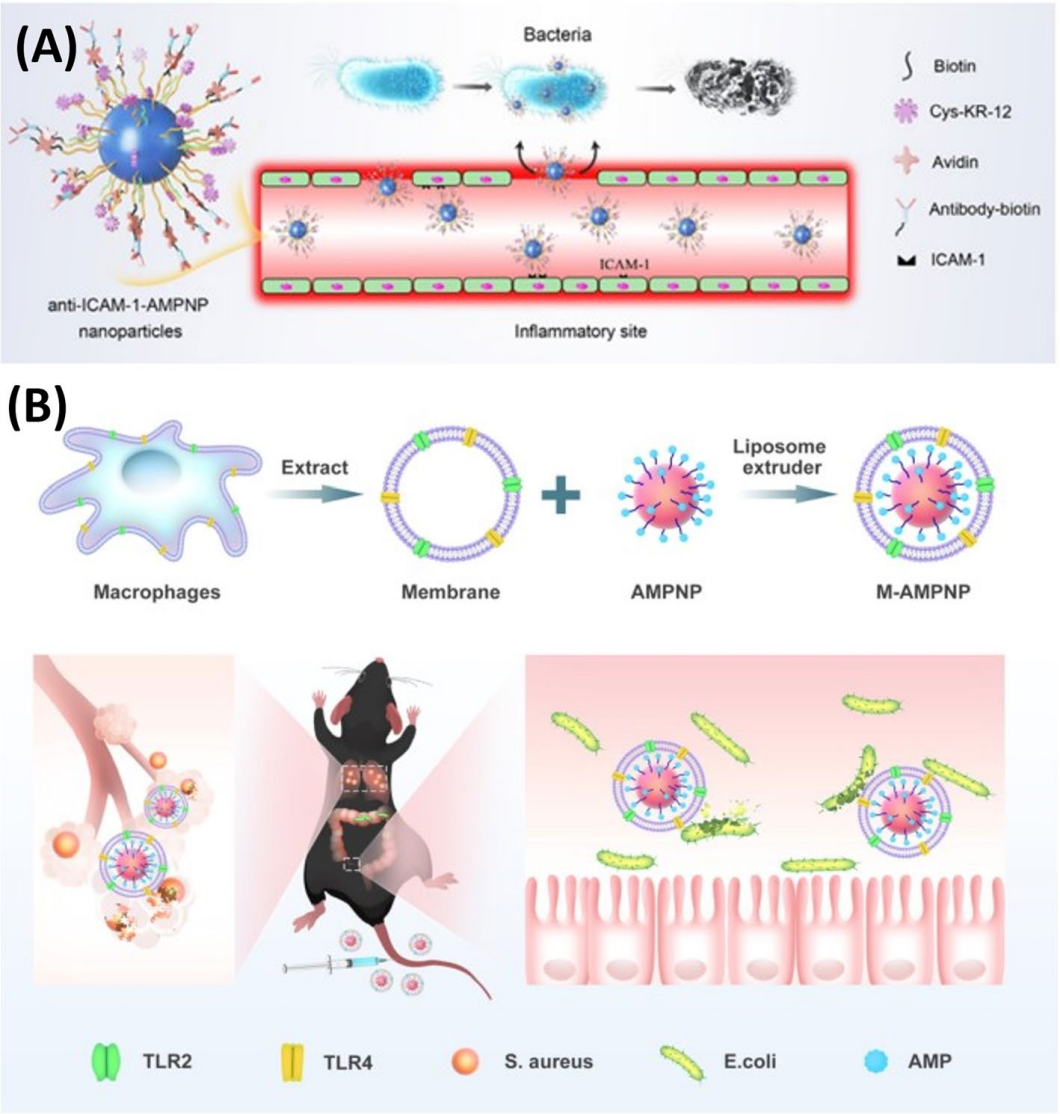
**A** Preparation of anti-ICAM-1-AMPNP to specifically target inflammation sites with overexpressed ICAM-1 receptor. **B** Preparation of macrophages membrane-coated AMPNP (M−AMPNP) to specifically target bacteria through the TLR2 and TLR4 on the macrophage membrane (Adopted from [22, 160])

Carrying on with the application of peptide nano-assembly, Pan and colleagues reported two studies [22, 160] on the development of co-assembled antibacterial peptide polymeric nanoparticles (AMPNP) for targeting bacteria and inflammation sites to combat bacterial sepsis. They synthesized antibacterial peptide (KR-12: KRIVKRIKKWLR)-grafted amphiphilic block copolymer and biotin grafted block copolymer, which were co-assembled in aqueous solution to produce the AMPNP. After that, as illustrated in Fig. 7, they tried two different approaches to achieve AMP-targeted delivery against sepsis. In their first approach [22], they modified the AMPNP with an antibody against intercellular adhesion molecule-1 (anti-ICAM-1 antibody) to target the inflammation sites with over-expressed ICAM-1 receptor. In their second targeting approach [160], they coated the AMPNP with a macrophage membrane of the mouse leukemia cells of monocyte-macrophage (M) to achieve specific binding to bacteria through the bacterial recognition molecules (Toll-Like receptors) on the macrophage membrane. The anti-ICAM-1-AMPNP and the M-AMPNP have shown specific targeting and adhesion to the inflamed human cells and bacterial cells, respectively. The in vitro antibacterial activity of the anti-ICAM-1-AMPNP and M-AMPNP was evaluated against *E. coli, S. aureus*, and MRSA, and they both exhibited good antibacterial efficacy. Moreover, when evaluated in vivo on mice sepsis model, both nanosystems have demonstrated a superior effect over the uncoated AMPNP regarding the reduction in serum cytokines (IL-1β, TNF-α, and IL-6) levels and inflammatory cells tissue infiltration.

Overall, both nanosystems (anti-ICAM-1-AMPNP and M-AMPNP) are promising and suggest a potential efficacy in bacterial sepsis management by explicitly targeting the inflammation sites and the causative bacteria. It is worth noting that, although the in vitro antibacterial activities of anti-ICAM-1-AMPNP and M-AMPNP were comparable to the bare peptide when evaluated in vivo*,* the nanosystems' efficacies were superior, which could be due to the improved stability of nanoparticles against proteases enzymes and the specific targeting and delivery to the inflammation and bacterial infections sites. However, using macrophage membranes derived from cancerous mouse cells could raise safety and immunogenicity concerns. Therefore, we think this mouse macrophage coating should be carefully considered when the nanosystem is to be further taken for clinical translation.

##### Nano-encapsulated AMPs

Encapsulation into nanosystems is another effective strategy for nano-delivery of AMPs to fight bacterial sepsis. This nano-encapsulation improves the AMPs' stability, reduces systemic toxicity, and improves their therapeutic efficacy [157]. In this context, Saúde et al. have encapsulated the antimicrobial peptide Clavanin A in a polymeric matrix for bacterial sepsis control, aiming to improve its stability and therapeutic efficacy [161]. The Clavanin A peptide (VFQFLGKIIHHVGNFVHGFSHVF-NH2) was nanostructured in a mixture of the methacrylate polymers EUDRAGIT® L 100-55 and EUDRAGIT® RS 30 D to make a nano-antibiotic. The nano-antibiotic demonstrated a sustained release, with 69% of the loaded Clavanin A released after 48 hrs. The in vitro antibacterial assay showed that nanoparticles containing 12 µg of Clavanin A inhibited the growth of *S. aureus* by 91%, *K. pneumoniae* by 20%, *P. aeruginosa* by 39.8%, and no effect on *E. coli*. In vivo efficacy of the nano-antibiotic evaluated on polymicrobial sepsis model on mice showed a 100% survival rate under a sub-lethal dose of bacteria and a 40% survival rate with a lethal inoculum. It is worth mentioning that, although the peptide loading significantly reduced the stimulation of pro-inflammatory cytokines (TNF-α, IL-12, and IL-10) release in vitro compared to the blank nanoparticles, these nanoparticles-induced cytokines releases were still significant. Therefore, we believe more in vivo evaluations are needed in this regard, and it would be better to avoid using these thiolated methacrylate polymers for antisepsis drug delivery as they are known to induce inflammation and cytokines release [168].

Another study carried out by Hassan and coworkers also reported the nano-encapsulation of an AMP (Mastoparan (Mast)) in polymeric nanoparticles to improve its stability and efficacy in managing multidrug-resistant bacterial sepsis [162]. They nano-encapsulated Mast (INLKALAALAKKIL-NH2) by structuring it with chitosan to produce a chitosan–Mast nano-construct (Mast-Cs NC). Mast-Cs-NC's in vitro antibacterial activity against *A. baumannii* clinical isolates demonstrated a significantly lower MIC of 4 μg/mL compared to the bare-Mast, which got an MIC of 16 μg/mL. When evaluated for in vivo efficacy on an *A. baumannii-*induced mice sepsis model, Mast-Cs-NC improved the physical activity and significantly decreased the blood bacterial counts compared to the chitosan and bare-Mast treated groups. Their findings showed enhanced in vitro and in vivo activity; however, they didn't report evaluation of the peptide release behavior from the nanosystem, which we believe is a fundamental property that will affect the selection of dosing frequency in clinical settings.

While the other studies reported encapsulation of functional AMPs in nanoparticles, Hou et al. have used an alternative unique approach by encapsulating the mRNA of the AMP-IB367 ( RGGLCYCRGRFCVCVGR_CONH2_) linked to mRNA of cathepsin B (CatB) (AMP-CatB mRNA) in vitamin C lipid nanoparticles (VLNPs) [163]. They transfected the nanoparticles in macrophages, where the mRNA will be translated to functional AMP-IB367 and CatB. CatB is an endogenous lysosomal protein that assists in translocating AMP-IB367 inside the macrophages' lysosomes, resulting in macrophages containing antimicrobial peptides linked to cathepsin B in the lysosomes (MACs). Upon the adoptive transfer of MACs to animals infected with bacteria, the lysosomes will fuse with phagosomes encapsulating bacteria and effectively kill the bacteria through both AMP-IB367 and lysosomal antimicrobial constituents. In vitro, MACs showed strong bacterial growth inhibition of 87% when evaluated against multi-drug resistant *S. aureus* (MDRSA) intracellular infection on RAW264.7 cells. MACs also significantly decreased bacterial loads in blood and improved survival rates of MDRSA-induced septic mice. Their findings presented the applicability of using nano-delivered mRNA of AMPs to target intracellular bacterial infections and sepsis. However, this strategy is limited by the possibility of mRNA degradation during loading and transfection, the immunogenicity that may arise from the adoptive transfer of macrophages, and the difficulties of scaling up these complex nanosystems. Therefore, we think these limitations must be carefully addressed before the MACs can be used in clinical practice.

##### AMPs conjugated to nanoparticles surface

Covalent conjugation of AMPs to the surface of nanoparticles has been applied to achieve nano-delivery of these conjugated AMPs. The conjugation can be accomplished on various types of nanoparticles, including both organic and metallic nanoparticles [24]. This approach is beneficial, especially in the cases of organic nanoparticles where another antisepsis drug can be encapsulated in the nanosystem and achieve simultaneous multiple drug delivery to target various pathways involved in the complex pathophysiology of sepsis. So far, only two studies have been reported on the covalent conjugation of AMPs to the surface of nanoparticles to enhance the stability and efficacy against bacterial sepsis, that is, one has conjugated the AMP to gold nanoparticles [164], and the other conjugated AMP to the surface of liposomes loaded with antibiotic [165]. Therefore, this strategy has not been thoroughly investigated, providing an opportunity to efficiently administer AMPs alone or combined with other drugs to address bacterial sepsis.

Rai and colleagues conjugated Cecropin melittin-cysteine (CM-SH: KWKLFKKIGAVLKVLC) AMP to gold nanoparticles (Au NPs) to improve the physiological stability and therapeutic efficacy against bacterial sepsis [164]. The optimized AMP-conjugated gold nanoparticles (CM-SH-Au NPs) were produced in one-step synthesis and found to have high AMP concentration (50% per nanoparticle mass). The in vitro antibacterial activity of CM-SH-Au NPs against *S. aureus* and *E. coli* showed a 4-fold reduction in MIC compared to the free CM-SH peptide. Unlike the free CM-SH peptide, CM-SH-Au NPs were found to be resistant to degradation, retaining even in the presence of cell culture media, human serum, and proteolytic enzymes such as trypsin, *S. aureus* V8 protease, and human neutrophil elastase. To evaluate the antimicrobial resistance development, they exposed *E. coli* to a sub-MIC dose of CM-SH-Au NPs for 28 days and then evaluated their efficacy against the treated strain. CM-SH-Au NPs were found to be still effective against the sub-MIC-exposed *E. coli* strain with no resistance development, unlike the control drug, chloramphenicol, which developed resistance after only 3 days of exposure. The therapeutic potential of CM-SH-Au NPs was also evaluated against CLP mice model of sepsis and demonstrated a significant reduction in bloodstream bacterial count and IL-10 level compared to unconjugated Au-NPs and free-CM-SH. We contend that their findings are promising and provide an effective strategy for improving the physiological stability and therapeutic efficacy of AMPs. However, the use of metallic nanoparticles needs comprehensive toxicity evaluations as they are known to carry more risk of systemic toxicity [169] than organic nanoparticles.

**Fig. 8:**
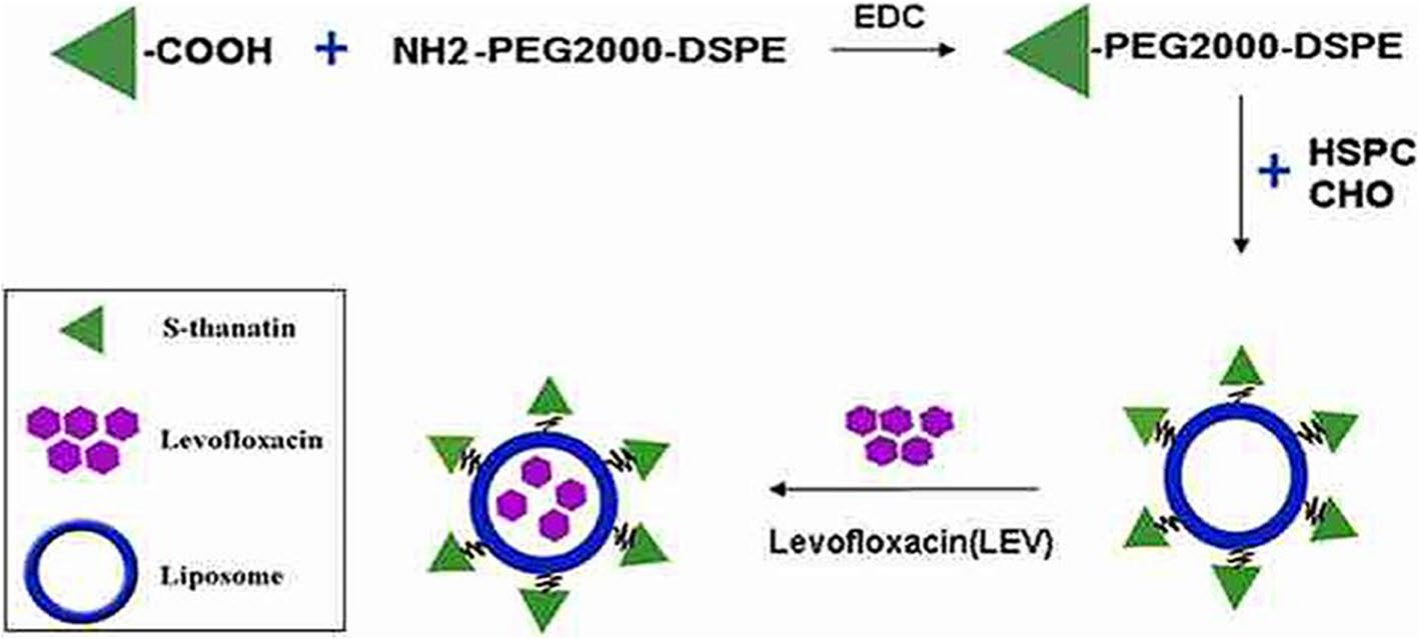
Preparation of Ts-LPs-LEV. Adopted from [165]

In another study, Fan et al. linked the AMP S-thanatin (Ts) (GSKKPVPIIYCNRRSGKCQRM) to the surface of liposomes loaded with levofloxacin (Ts-LPs-LEV) to target *K. pneumoniae* induced sepsis [165]. As illustrated in Fig. 8, Levofloxacin-loaded liposomes were prepared by thin film hydration method with the incorporation of Ts-PEG2000-DSPE to produce positively charged liposomes and Ts anchored on the surface. The incorporation of Ts synergistically improved levofloxacin's antimicrobial activity on sensitive *K. pneumoniae* and restored its sensitivity on multidrug-resistant clinical isolates. The calculated MICs of Ts-LPs-LEV were 2 to 8 folds less than that of LPs-LEV on different strains of *K. pneumoniae.* Moreover, when evaluated on mice sepsis model of MDR *K. pneumonia* clinical isolate, the anchoring of Ts peptide resulted in a significant difference in bacterial clearance from blood and mice's survival rates with a reduction in lethality from 73.3% to 6.7% compared to LPs-LEV. It should be pointed out that while the peptide linking synergistically improved the efficacy of LEV, the Ts-linked liposomes without loading of LEV showed no activity against the majority of tested bacterial isolates (16 out of 18 isolates). However, the free peptide was effective on those isolates; therefore, we think this peptide's activity loss needs to be addressed and investigated. Furthermore, they didn't examine the enzymatic stability of conjugated peptide compared to the free peptide, which we believe is one of the main advantages of incorporating AMPs into nanosystems. Besides, they didn't evaluate the release pattern of the Ts and LEV, which is critical in determining dosing frequencies in clinical settings.

In a nutshell, nano-delivery of AMPs has been proven to be an effective strategy to overcome the shortcomings of antimicrobial peptides, such as physiological instability and systemic toxicity, resulting in improved biosafety and efficacy against bacterial infections and sepsis both in vitro and in vivo. The AMPs have been nano-delivered through various strategies, including conjugation with hydrophobic moieties to allow their self-assembly into nanoparticles, encapsulation in organic nanosystems, and linking the surfaces of metallic and organic nanoparticles. Therefore, we believe that with more investigations, these nanomedicines could be scaled up and become available for clinical use to help combat the life-threatening bacterial sepsis and the growing antimicrobial resistance.

#### Anti-inflammatory peptides nanosystems

Recently, anti-inflammatory peptides (AIPs) nanosystems have shown excellent properties, making them exceptional therapeutic candidates for sepsis management [23, 170]. These characteristics include potent neutralizing effects against pro-inflammatory molecules [171–173], improved biodegradability and biocompatibility [174], advanced delivery properties [175], and multiple actions across various intracellular inflammatory pathways [30, 176]. This section covers all AIP nanosystems reported for sepsis management, including self-assembled nanostructured AIPs and other nanocarriers used to deliver AIPs.

##### Self-assembled nanostructured AIPs

Self-assembled nanostructured peptides have been introduced to improve the anti-inflammatory properties of LPS-binding proteins (Limulus anti-LPS factor, serum amyloid P, and bactericidal permeability-increasing protein) by Mas-Moruno et al. This study successfully synthesized and structurally characterized several N-acylated peptides derived from the above proteins with advanced anti-inflammatory activity against LPS-induced cytokines storm. In vitro investigations have been done to evaluate their biosafety profile against RAW 264.7 macrophages, and most of them were found to be biosafe and tolerable. Compared to their parent peptides, some N-acylated peptides showed up to 10-fold enhancement in the in vitro LPS neutralizing activity within their biosafe concentration ranges. This activity enhancement may be related to their ability to form fibril-like and micellar nanostructures, as shown during TEM imaging. Their findings are promising, and we suggest they can be taken for preclinical and clinical evaluations to prove these results and allow for application in clinical settings [171].

Later, Tram et al. designed a stimuli-responsive self-assembled nanostructured dual active peptides (anti-inflammatory and antimicrobial) as an effective antisepsis agent with an advanced ability to form amyloid-like nanostructured nets in response to LPS and other bacterial endotoxins contact [173]. These multifunctional positively charged synthetic β-hairpin peptides could efficiently exert their anti-inflammatory effect through selective entrapping of negatively charged pro-inflammatory molecules and cytokines such as TNF-α, IL-6, LPS, and lipoteichoic acid (LTA) and, therefore, inhibiting bacterial endotoxin-induced cytokine storm. On the other hand, they exerted their antimicrobial activity by physically trapping bacterial cells and lysing bacterial membranes through interaction with the negatively charged bacterial cell walls and membranes. In this study, the selected peptides have shown promising bacterial toxin-neutralizing activity, which has been evaluated in a bacterial toxin-challenged-murine macrophage cell line model (in vitro) and acute lung injury mice model (in vivo). Furthermore, this study has involved various techniques to show the selective trapping of negatively charged pro-inflammatory cytokines (TNF-α and IL-6). In summary, modifications that confer AIPs the ability to self-assemble into nanostructured systems significantly enhanced their neutralizing activity against pro-inflammatory molecules and overall antisepsis outcomes with improved stability and circulation time.

##### Conventional nanocarriers-delivered AIPs

Several nanocarriers, including polymeric-based [30, 176], protein-based [174], and metallic-based nanosystems [172], have been employed to deliver AIPs in attempts to improve their antisepsis activity. Table 3 summarizes the studies reported on designing AIP-loaded nanocarriers for sepsis management, highlighting the utilized AIP, the type of nanocarrier, the preparation method, and the key advantages of AIP nano-delivery. As depicted, nano-delivery enhanced the peptide's anti-inflammatory activity, stability, circulation time, biodistribution, biodegradability, and hemocompatibility. Moreover, nanocarriers are involved in the AIPs' co-delivery with antibiotics and other antithrombotic peptides to develop innovative and comprehensive antisepsis therapies with promising clinical outcomes. The studies will be discussed in this subsection based on the type of constituents of the nanocarriers, including polymeric, metallic, and protein-based materials.

**Table 3:** List of anti-inflammatory peptides conventional nanocarriers

**AIP**	**Noncarrier**	**Preparation method**	**Key advantages**	**Reference**
GLP-1 LP17	PEG-based Micelles	Emulsification	Peptides stabilization on their active form and Enhanced bioactivity	[176]
MB2mP6 (Myr-FEKEKL)	PEG-based High-loaded peptide nanoparticles	Film rehydration	Enhanced bioactivity	[30]
Thymulin	Acrylate-based nanoparticles	Coprecipitation	Enhanced bioactivity and circulation time elongation	[177]
Agglutinating salivary proteins-derived peptides	Magnetic iron oxide Nanoparticles	Coprecipitation	Enhanced bioactivity	[178]
PBP10	Metallic iron oxide Nanoparticles	Coprecipitation	Enhanced bioactivity and improved hemocompatibility	[172]
GF9	Ferritin-based nanocage	Emulsification	Enhanced bioactivity, circulation time elongation, and improved biocompatibility	[174]

*GLP-1* Human glucagon-like peptide, *LP17* Triggering receptor expressed on myeloid cells 1 (TREM-1) inhibitor peptide, *PEG* Polyethylene glycol, *PBP10* Pro-inflammatory molecules-neutralizing rhodamine B-conjugated peptide, *GF9* TREM-1-specific inhibitory peptide

Firstly, modified polyethylene glycol and acrylate polymers have been used to fabricate biocompatible nanocarriers to improve AIPs delivery for sepsis treatment [30, 176, 177]. These polymer-based nanocarriers significantly increased the antisepsis activity of AIPs by enhancing their stability, anti-inflammatory activity, and circulation time. For instance, Sadikot et al. utilized distearoyl phosphatidyl-linked PEG (DSPE-PEG_2000_) to form 15 nm-micelles as an innovative co-delivery system for the two AIPs; human glucagon-like peptide (GLP-1) and triggering receptor expressed on myeloid cells 1 (TREM-1) inhibitor peptide (LP17)) to treat sepsis-related acute lung injury. This phospholipid micellar system stabilized both peptides in their activated alpha helix form and conferred prolonged circulation and in vivo bioactivity time compared to their parent peptides. Similarly, Cheng et al. and colleagues used DSPE-PEG_2000_ to develop highly loaded peptide nanoparticles to deliver anti-inflammatory/antithrombotic dual active peptide, MB2mP6 (Myr-FEKEKL), as a new thoughtful approach for sepsis treatment [30]. MB2mP6 nanoparticles effectively inhibited both thrombosis and inflammation with limited vascular leakage by targeting inflammatory and thrombotic pathways associated with integrin’s G-protein alpha subunit-13 (Gα13) interactions in leukocytes and platelets. Immediate and late administration of MB2mP6 nanoparticles after severe sepsis initiation significantly increased mice survival rate, reduced inflammatory and thrombosis mediators, and prevented tissue and organ damage.

Likewise, utilizing polymeric material for AIP delivery, Novoselova et al. used polyacrylate-modified polybutyl cyanoacrylate polymer to formulate thymulin (an AIP thymic peptide)-bound nanoparticles to efficiently treat chronic inflammation and sepsis [177]. The developed acrylate-based nanoparticles showed 90% entrapment efficiency, improving the delivery aspect of thymulin against sepsis, such as circulation time and biodegradability. Thymulin-loaded nanoparticles efficiently alleviated sepsis-induced cytokines storm, decreased heat shock proteins and TLR-4 expression, reduced apoptosis, and increased splenic cell counts in mice. In summary, the findings of the three studies [30, 176, 177] demonstrated that utilizing polymeric nanosystems significantly enhanced the therapeutic efficacy of AIPs. However, it is essential to highlight that they didn't report much characterization of the developed nanosystems, such as size, PDI, ZP, and release kinetics measurements, which are highly important in indicating nanomedicines' storage stability and dosing frequencies.

Secondly, two studies by Karawacka et al. and Piktel et al. have used magnetic metallic nanoparticles to immobilize anti-inflammatory peptides via electrostatic interaction to improve their stability, activity, and circulation time [172, 178]. In their study, Karawacka and colleagues used a coated superparamagnetic iron oxide nanoparticle to bind and immobilize agglutinating salivary proteins-derived peptides (LPS-neutralizing peptides) via hetero functional linkers. This modification significantly enhanced the in vitro LPS-neutralizing activity of conjugated peptides by more than 3-fold when evaluated by the endotoxin binding assay [178]. In the second study, Piktel and coworkers developed an innovative iron oxide-based peptide nanosystem with advanced delivery properties. Synthetic pro-inflammatory molecules-neutralizing peptide PBP10 (synthetic rhodamine B-conjugated peptide, bioinspired from the naturally occurring protein human plasma gelsolin) and its derivatives were used in this study to functionalize iron oxide nanoparticles. These fabrications simultaneously enhanced the antibacterial activity of metallic nanoparticles, the anti-inflammatory properties of immobilized peptides, and their biocompatibility properties, promoting the promising potential of AIPs metallic nanosystems as effective antisepsis therapeutic agents [172].

Finally, ferritin-based nanocages are well-known nanocarriers that improve stability and overall activity for different types of drugs due to their excellent properties, such as inherent cavity sizes and biocompatibility [179]. With this regard, Wei and coworkers used an emulsification technique to formulate ferritin-based nanocages as an efficient nano drug delivery system for both anti-inflammatory peptide GF9 (a TREM-1 inhibitor) and the antibacterial agent streptomycin as a dual therapy against bacterial-induced sepsis [174]. Using an *E. coli*-induced sepsis mice model, this Antibacterial/ anti-inflammatory co-delivery successfully reduced bacterial burden, suppressed harmful inflammatory responses, prevented lungs from sepsis-associated tissue damages, and achieved better overall clinical outcomes and survival rates compared to monotherapies. Thus, we believe ferritin-based co-delivery of AIPs with antibacterial agents could be an efficient therapeutic strategy against sepsis.

##### Stimuli-responsive nanocarriers- delivered AIPs

Compared to conventional nanocarriers, stimuli-responsive nanocarriers have shown superior clinical outcomes due to their advanced release patterns that accumulate the loaded therapeutic agents in their site of action in response to distinguished pathophysiological changes [180]. Concerning this, a brilliant study by Lee et al. reported stimuli-responsive ferritin-based nanocages that simultaneously delivered two bioactive peptides, targeting two different intracellular pathways to improve sepsis control and reduce harmful side effects [175]. In this study, ferritin was Genetically modified by inserting the endothelial protein C receptor-targeting ligand (PC-Gla domain) and protease-activated receptor-1 activator (TRAP peptide) to form ferritin-based nanocarriers, which showed an advanced antisepsis activity. This promising activity has been further improved by inserting a matrix metalloproteinase-sensitive linker to confer PC-Gla domain a stimuli-response release pattern in response to metalloproteinase at the metalloproteinase-rich inflammatory sites. In vitro and in vivo sepsis models confirmed the stimuli-responsive PC-Gla release, significantly reducing inflammatory cells infiltration and lung injury scores and improving mice survival rates after CLP.

**Fig. 9:**
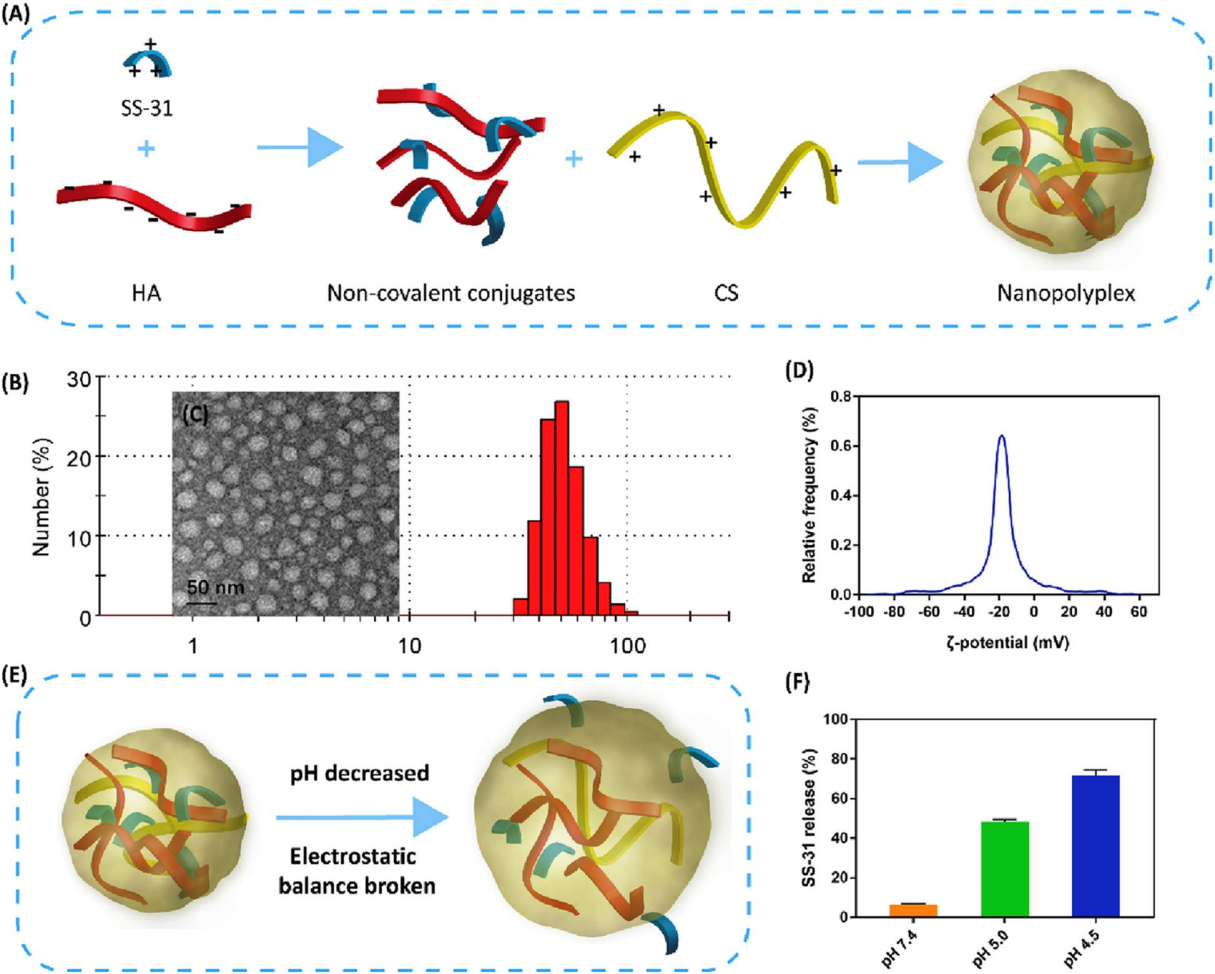
Preparation and evaluation of SS-31 loaded nanoplexes. **A** The method for fabricating nanoplexes through electrostatic complexation; (**B**) and (**D**) DLS characterization; (**C**) TEM imaging; (**E**) pH-responsiveness; (**F**) release patterns at different pHs. (Taken from [181])

Later, Lui et al. reported pH-responsive nanoplexes that could target CD44-overexpressed cells as an efficient stimuli-responsive nanocarrier of SS-31 peptide (an AIP) against sepsis-induced acute kidney injury [181]. This innovative design perfectly overcame the poor pharmacokinetic characteristics of the loaded peptide SS-31, enhancing its activity and targetability. As presented in Fig. 9, biocompatible polymers Hyaluronic acid and chitosan (CS) electrostatically interacted to form stable nanoplexes, promoting payload release via low pH condition destabilization. SS-31 loaded nanoplexes were stable at physiological pH with an average size of 53 nm, ZP of -20 mV, and PDI of 0.17. large surface charge conferred nanoplexes high stability to prevent aggregation and enhance accumulation at the site of action. In vitro drug release studies confirmed the pH-responsive release pattern with an approximately 10-fold higher drug release percentage at pH 4.5 compared to pH 7.4. SS-31 loaded nanoplexes showed an enhanced intracellular uptake and higher antioxidant and antiapoptotic properties compared to bare SS-31 peptides in both in vitro and in vivo studies. Furthermore, histopathological analysis revealed that treatment with SS-31-loaded Nanoplexes improved kidney functions and reduced sepsis-associated tissue damage and tubular injury.

In conclusion, exploring nano-delivered AIPs to combat sepsis has promising outcomes. The improved neutralizing action of AIPs in nanosystems against pro-inflammatory cytokines opened avenues for further preclinical and clinical evaluations. In addition, as compared to free peptides, nano-delivered AIPs exhibited markedly enhanced stability, circulation time, and therapeutic effectiveness. Furthermore, stimuli-responsive nanocarriers demonstrated superior clinical outcomes with advanced release patterns, offering a targeted and efficient approach to sepsis treatment.

Overall, the studies discussed in the above two sections underscore the potential of nano-delivery systems to enhance bioactive peptides (AMPs and AIPs)' stability and therapeutic efficacy for effectively managing sepsis, presenting a compelling foundation for further exploration and clinical translation.

#### Peptides as nanocarriers components

##### Peptides as targeting moieties on nanoparticles’ surface

The surface of nanoparticles can be modified using various techniques and modifying materials. This surface modification achieves several advantages, including targeted and site-specific drug delivery, improved cellular uptake of loaded drugs, and enhanced nanosystems' physicochemical, biological, and therapeutic properties [44]. Different materials, such as antibodies [182], oligonucleotides [183], polymers [184], and peptides [185], have been used for nanoparticles’ surface modifications. Table 4 presents a summary of peptides’ application for surface modification of nanoparticles delivered against bacterial sepsis, showcasing the type of the nanosystem, peptide sequence, biological target, loaded drugs, key characterizations, and key findings. As presented, peptides have been used as targeting moieties for specific delivery to inflammation sites, bacterial cells, and body organs. It is obvious that the most studied application was the use of peptides to target inflammation sites through the overexpressed receptors, enzymes, and other proteins. Within these, ICAM-1 was the most targeted inflammatory site components. This open avenues for more research on the application of peptides to target other sepsis inflammatory-microenvironment’s overexpressed components such as selectins, CD44, TREM-1, and protease-activated receptor-1 (PAR-1) [186]. Also, the of targeting invading bacteria and specific body organs that are at high risk of damage during sepsis are yet to be more explored. The studies will be discussed in the following subsections based on the biological target that has been utilized to achieve improved sepsis management.

**Table 4:** Summary of peptides utilization as targeting moieties on the surface of nanosystems to combat bacterial sepsis

Nanosystem	Peptide sequence	Biological target	Loaded drug	Key characterization	Key findings	Reference
PLGA nanoparticles	cyclo(1,12)PenITDGEATDSGC (cLABL) peptide	ICAM-1	NA	DLS Cellular binding and uptake.	Size: 244 ± 15 nm; PDI: 0.215; ZP: -23.3 ± 1.0 mV. Superior binding and internalization into interferon -γ-treated HUVECs cells.	[187]
PLGA nanoparticles	γ3 peptide (NNQKIVNLKEKVAQLEA)	ICAM-1	SFX TAC	TEM DLS LC & EE% In vitro drug release. Cellular uptake. In vitro and In vivo efficacy.	Shape: Spherical; size: 183.7 ± 9.4 nm; ZP: -40 mV; LC: (SFX: 15.47 ± 0.91, TAC: 20.22 ± 1.16); EE%: (SFX: 50.73± 4.09, TAC: 63.39 ± 3.52). Sustained drug release. 2 to 3 times more uptake by TNF-α activated cells Superior in vitro and in vivo antibacterial and anti-inflammatory efficacy.	[188]
PLGA nanoparticles	γ3 peptide (NNQKIVNLKEKVAQLEA)	ICAM-1	Ciprofloxacin	TEM DLS In vitro drug release. Cellular uptake. In vitro and In vivo efficacy.	Shape: core-shelled nanoparticles; size: 157 nm; ZP: -22 mV. Sustained drug release. Superior uptake by TNF-α activated cells. Superior in vitro and in vivo antibacterial and anti-inflammatory efficacy.	[189]
Liposomes	RGD	Integrin	Curcumin	TEM DLS LC & EE% Cellular Targeting Assay. Antipyroptosis Assay. In vitro ROS scavenging. In vivo efficacy.	Shape: disc-like; size: 41.95 ± 19.85 nm; PDI: 0.228 ± 0.127; LC: 22.2 ± 1.4%; EE%: 99.9 ± 1.1%. Superior uptake by LPS-stimulated RAW264.7 cells. Significant in vitro reduction in intracellular ROS and inhibition of pyroptosis. Remarkable inhibition of cytokines release (TNF-α and IL-6) and organ damage in mice.	[190]
HMPDA	Cys-LSA peptide (CLSALTPSPSWLKYKAL)	Dipeptidase 1	BAPTA-AM NAD^+^	DLS LC & EE% Intracellular Energy Homeostasis restoration. In vivo accumulation and antisepsis efficacy.	Size: 75.3 nm; ZP: -2.1 mV; LC: (NAD^+^: 23.8%, BAPTA-AM: 38.3%); EE%: (NAD^+^: 20.5%, BAPTA-AM: 57.9%). Superior restoration of mitochondrial function, intracellular Ca^++^ hemostasis, and antioxidant activity. Superior accumulation in liver, kidney, and lung of mice with LPS-induced sepsis. Prevention of organ damage with improved survival rates in septic mice.	[191]
MSN	WLSEAGPVVTVRALRGTGSW	Primary cardiomyocytes	L-arginine	TEM DLS In vitro drug release. In vitro cellular uptake. In vivo biodistribution and efficacy.	Shape: spherical; size: 186.67 nm; ZP: -6.23 mV. LIFU-responsive release: increased from 11.5% to 25.6% at pH 7.4. Higher affinity and localization at cardiomyocytes compared to other cells. Significantly higher distribution and accumulation in mice's hearts than in other organs. Effective prevention of sepsis-induced cardiac injury in mice.	[192]
Zeolite imidazolate framework-8 nanoparticles	KCSAVPLC	Kidney cells	FGF21	TEM DLS In vitro drug release. Cellular uptake. In vitro cytoprotective effect. In vivo biodistribution and efficacy.	Shape: polyhedral; size: 120.1 ± 5.4 nm; ZP: -17.4 ± 1.4 mV; pH-responsive drug release. Significantly higher in vitro uptake by renal cellular. Superior in vitro antioxidant, anti-inflammatory, and antiapoptotic efficacy on renal cells. Preferable accumulation in kidneys and better recovery of renal function in LPS-induced septic mice.	[193]
GBPs	D-/L-Cys-Phe (CF) dipeptide	Bacterial cells	NA	TEM and SEM. Photothermal properties assay. Bacterial cell affinity assay. In vitro and In vivo efficacy.	Shape: a spike shape (sea cucumber-like morphology); size: (Length: 200 ± 5.3 nm; width: 50 ± 2.8 nm). Higher bacterial cell affinity with the D-CF coating gave higher adsorption than L-CF. Significantly improved in vitro and in vivo efficacy.	[194]

*ICAM-1* Intercellular Adhesion Molecule-1, *DLS* Dynamic light scattering, *PDI* Polydispersity index, *ZP* Zeta potential, *HUVECs* Human umbilical cord vascular endothelial cells, *SFX* Sparfloxacin, *TAC* Tacrolimus, *TEM* Transmission electron microscopy, *LC* Loading capacity, *EE%* Encapsulation efficiency, *TNF-α* Tumor necrosis factor-α, *ROS* Reactive oxygen species, *IL-6* Interleukin-6, *HMPDA* Hollow mesoporous polydopamine nanocarrier, *BAPTA-AM* BA-AM, *O* O′-bis(2-aminophenyl) ethylene glycol-N,N,N′,N′-tetra acetic acid, tetra acetoxymethyl ester, *NAD*^*+*^ Nicotinamide adenine dinucleotide, *LPS* Lipopolysaccharides, *MSN* Mesoporous silica nanoparticles, *LIFU* Low-intensity focused ultrasound, *FGF21* fibroblast growth factor 21, *GBPs* Chiral gold nano-bipyramids, *D-CF* Dextro isomer of Cys-Phe dipeptide, *L-CF* Levo isomer of Cys-Phe dipeptide

##### Inflammation sites targeting peptides

Inflammation sites, such as those of sepsis, are characterized by an overexpression of certain proteins involved in the inflammation processes [188, 191]. These up-regulated proteins can be exploited to develop smart nanosystems modified with targeting moieties (such as peptides) that bind specifically to these proteins, resulting in targeted drug release at the inflammation sites [195]. In this subsection, we discuss the utilization of peptides as surface modifiers for nanosystems to target various upregulated proteins at the sepsis’s inflammation sites. The studies will be organized according to the targeted protein.

ICAM-1 is a key adhesion molecule on epithelial cells that acts as a ligand to the integrins receptors on polymorphonuclear leukocytes, mediating their recruitment and migration into tissues. ICAM-1 is typically expressed at low levels; however, its expression is up-regulated during sepsis and inflammatory conditions, increasing the leukocytes adhesion [196]. Therefore, ICAM-1 targeting ligands such as peptides [187] and anti-ICAM-1 antibodies [22] have been used to modify nanoparticles to achieve inflammation sites targeted drug release. To this end, three studies [187–189] have reported the use of peptides for surface modification of Poly DL-lactic-co-glycolic acid (PLGA) nanoparticles targeting the inflammation-induced overexpression of ICAM-1. In their study, Zhang group reported a proof-of-concept investigation in which they decorated PLGA nanoparticles with cyclo (1,12) PenITDGEATDSGC (cLABL) peptide, which is proven to have a good binding affinity to the D1 domain of ICAM-1 [197], to target the overexpressed ICAM-1 on inflamed cells. cLABL-PLGA-NPs demonstrated rapid binding and internalization into human umbilical cord vascular endothelial cells (HUVECs) with up-regulated ICAM-1 induced by interferon -γ treatment. They found the binding of cLABL-PLGA-NPs to be inhibited by pretreatment with free cLABL, proving that the binding of nanoparticles was due to the peptide conjugation [187].

**Fig. 10:**
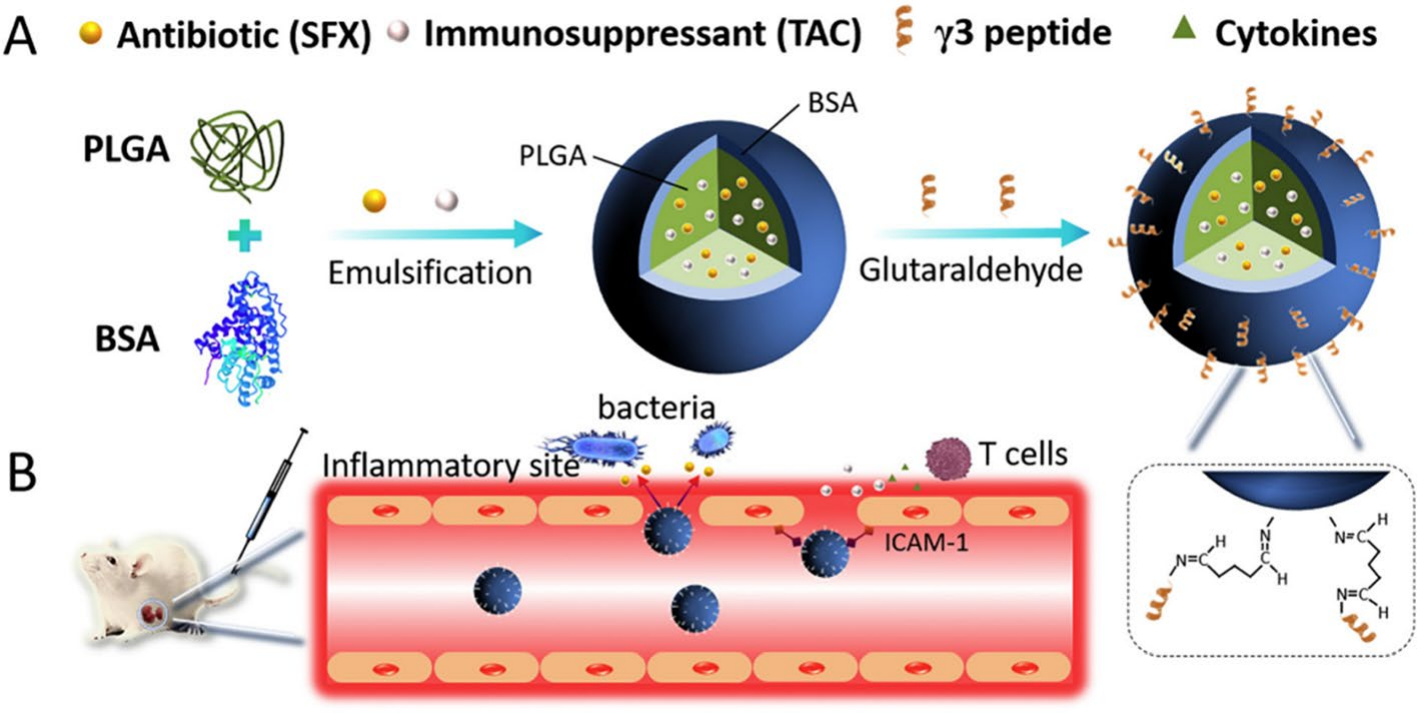
Preparation and in vivo evaluation of γ3-PLGA NPs (**A**) Preparation of γ3-PLGA NPs loaded with Sparfloxacin and Tacrolimus (**B**) Effective treatment of lung-infected mice by specific targeting of the overexpressed ICAM-1 (Taken from [188])

The other two studies by Yang et al. and Liu et al. utilized γ3 peptide (NNQKIVNLKEKVAQLEA) as an ICAM-1 ligand for surface modification of PLGA nanoparticles achieving targeted release of the loaded antisepsis agents at the sepsis’s inflamed sites. Yang and colleagues, as illustrated in Fig. 10, utilized the γ3-PLGA-NPs for co-delivery of the antibiotic Sparfloxacin (SFX) and the anti-inflammatory/immunosuppressant Tacrolimus (TAC), resulting in γ3-PLGA-NPs@SFX/TAC. Conversely, Liu et al. applied a monotherapy strategy by loading only ciprofloxacin (CIP) in the γ3 modified PLGA-NPs. However, Liu’s group coated the PLGA-NP with red blood cells (RBC) membrane producing γ3-RBCNPs@CIP to avoid immune vigilance and provide prolonged circulation time. Both γ3-PLGA-NPs@SFX/TAC and γ3-RBCNPs@CIP showed good cytocompatibility, hemocompatibility, and low systemic toxicity. When evaluated on TNF-α activated HUVECs cell line, both nanosystems demonstrated significantly higher binding to the stimulated cells than the peptide-unconjugated nanoparticles. Furthermore, they both exhibited superior in vitro antibacterial efficacy.

In the in vivo therapeutic efficacy evaluated against acute lung infection mice models, both γ3-PLGA-NPs@SFX/TAC and γ3-RBCNPs@CIP achieved significantly higher lung tissue accumulation and significantly reduced the bacterial load, inflammatory cytokines level, and inflammatory cells infiltration. As a result, they improved the mice survival rates compared to the peptide-unmodified nanosystems, proving the enhanced release at the inflamed lung tissues due to the ICAM-1 targeting. Both nanosystems have been extensively characterized and evaluated in vitro and in vivo. It should be noted that while the nanosystem developed by Yang group was assessed against both Gram-positive and Gram-negative bacteria (*S. aureus and P. aeruginosa*), the efficacy of the one by Liu et al. was studied only against Gram-negative bacteria* (K. pneumoniae).* On the other hand, Liu’s group proved the ability of their system to avoid macrophages’ phagocytosis, which we consider an essential feature that improves and prolongs the in vivo activity. Overall, we believe using peptides for surface modification of nanoparticles as targeting ligands to the overexpressed ICAM-1 at the inflammatory sites is a promising strategy for effective sepsis management with minimized systemic toxicities, as evidenced by these reports.

Exploring the same ICAM-1/integrin ligand-receptor pair, Shi and coworkers have also designed a peptide-modified nanosystem for inflammation targeting. However, their approach targeted the integrin receptor on the immune cells instead of the ICAM-1 ligand. They anchored the Arginine-Glycine-Aspartic Acid (RGD) peptide, a well-known integrin receptor ligand, on curcumin (Cur)-loaded liposomes (RGD-lipo/Cur) to achieve macrophages targeted Cur release against sepsis-induced inflammation [190]. While there was no significant increase in the uptake of RGD-unmodified liposomes after LPS-stimulation, RGD-lipo/Cur demonstrated a significantly higher uptake by LPS-stimulated RAW264.7 cells compared to the unstimulated cells. Also, the fluorescence microscope imaging showed colocalization of the RGD-lipo/Cur and the fluorescently labeled integrin receptors, indicating the liposomes' internalization to be through the RGD/integrin interaction. As a result, RGD-lipo/Cur showed a superior reduction in intracellular ROS levels and significantly inhibited the high inflammatory lytic programmed cell death (pyroptosis) of LPS-activated RAW264.7 cells compared to the peptide-unmodified liposomes. In vivo, RGD-lipo/Cur significantly reduced the release of inflammatory cytokines (TNF-α and IL-6) and prevented sepsis-induced organ damage in the LPS-induced mice sepsis model. Their findings showed the promising potential of RGD modification of Cur-loaded liposomes to improve sepsis management. However, they did not report ZP measurement, which we believe is a critical property in determining the storage and physiological stability of the nanosystem.

**Fig. 11:**
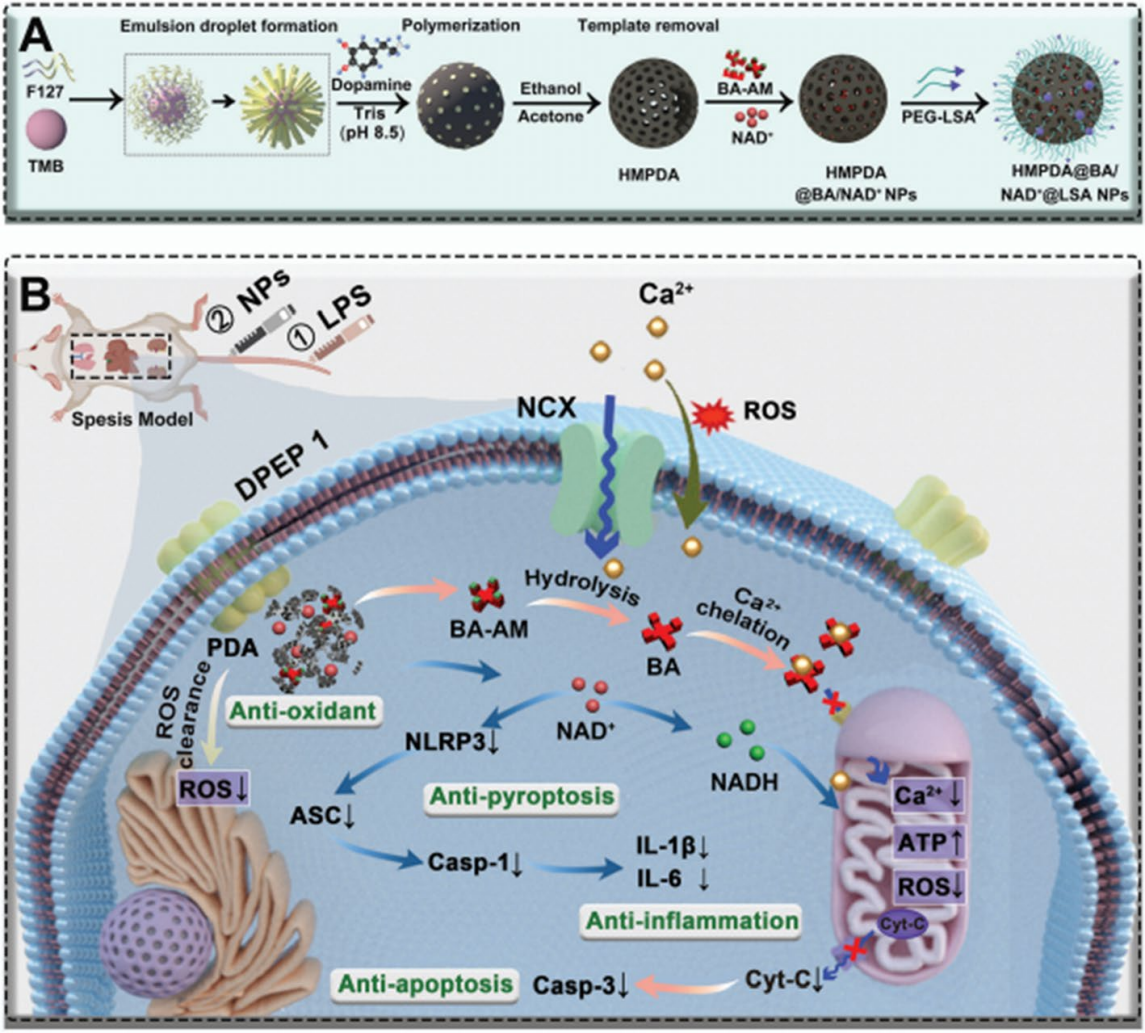
Preparation and mechanism of action of HMPDA@BA/NAD^+^@LSA NPs (**A**) Preparation of HMPDA@BA/NAD^+^@LSA NPs. **B** Pharmacological effects of HMPDA@BA/NAD^+^@LSA NPs in an LPS-induced sepsis mice model (Adopted from [191]

Another up-regulated protein during sepsis inflammatory conditions is dipeptidase 1 (DPEP1), expressed on cells of key organs such as the kidney, liver, and lung. Similar to ICAM-1, DPEP1 is an adhesion molecule for the recruitment of leukocytes [198, 199]. Therefore, DPEP1 ligands can be utilized for nanoparticles surface modification to accomplish targeted drug release at the inflamed tissues during sepsis. In this respect, Yan and colleagues exploited Cys-LSA peptide (CLSALTPSPSWLKYKAL), a DPEP1 ligand, for surface coating of hollow mesoporous polydopamine nanocarrier (HMPDA) specifically targeting inflammation sites for the management of sepsis. As depicted in Fig. 11, HMPDA was prepared by soft template method, loaded with NAD^+^ and BAPTA-AM, and grafted with LSA peptide to give HMPDA@BA/NAD^+^@LSA NPs [191].

Their approach was based on a triple therapy to prevent sepsis-induced cells, tissues, and organs damage with NAD^+^ restoring the energy production and exerting anti-inflammatory effect, BAPTA-AM chelating the overloaded intracellular Ca^++^ and PDA scavenging the intracellular ROS. When evaluated in vitro using H_2_O_2_-stimulated liver and kidney cells, HMPDA@BA/NAD^+^@LSA NPs demonstrated superior ability to restore mitochondrial function, Ca^++^ hemostasis, and antioxidant system and so rescued endangered cells. Studied in LPS-induced mice sepsis model, the accumulation of the peptide-modified nanoparticles in the key organs was markedly higher than that of healthy mice. Moreover, peptide modification significantly increased the accumulation of nanoparticles in the liver, kidney, and lung (1.4, 1.6, and 1.5 times) compared to the peptide-unmodified nanoparticles, proving the targeting of the overexpressed DPEP1 at the inflamed organs. As a result, HMPDA@BA/NAD^+^@LSA NPs significantly reduced the sepsis-induced key organs damage and improved the survival rate of mice. We argue that this multifunctional nanosystem with inflammation-targeted drug release is a promising nanomedicine to help combat sepsis’s multiple pathogenesis with less systemic drug exposure.

In conclusion, these studies underscore the potential of utilizing peptides for surface modification of nanoparticles, serving as selective targeting moieties for the up-regulated proteins at inflammation sites for sepsis management. However, more avenues are available to explore other up-regulated proteins using various peptide sequences.

##### Organ targeting peptides

Among the strategies for incorporating peptides as targeting moieties on nanosystems surface, a notable approach is targeting specific organs’ tissues and cells. Organ-specific peptides allow for accurate drug delivery to particular organs during sepsis, offering a mechanism to rescue key organs at high risk of damage and failure [192, 200]. In this regard, Huang et al. engineered zeolite imidazolate framework-8 nanoparticles coated with renal tubular epithelial cell membrane and modified with a kidney targeting peptide (KCSAVPLC) to promote specific drug uptake by renal tubular cells. This nano-construct, denoted as KMZ@FGF21, was loaded with the antioxidant/anti-inflammatory hormone, fibroblast growth factor 21 (FGF21), against sepsis-induced acute kidney injury (AKI) [193]. Peptide modification significantly increased the nanoparticles uptake by murine renal tubular epithelial cells (TCMK-1) in vitro and decreased intracellular oxidative stress, inflammation, and apoptosis compared to the peptide-unmodified nanoparticles. Moreover, when injected into mice, the fluorescently labeled KMZ@FGF21 favorably accumulated in kidney tissues. When evaluated in a sepsis-induced AKI mice model, KMZ@FGF21 exhibited superior antioxidant and anti-inflammatory efficacy, alleviated AKI, and improved renal function recovery.

**Fig. 12:**
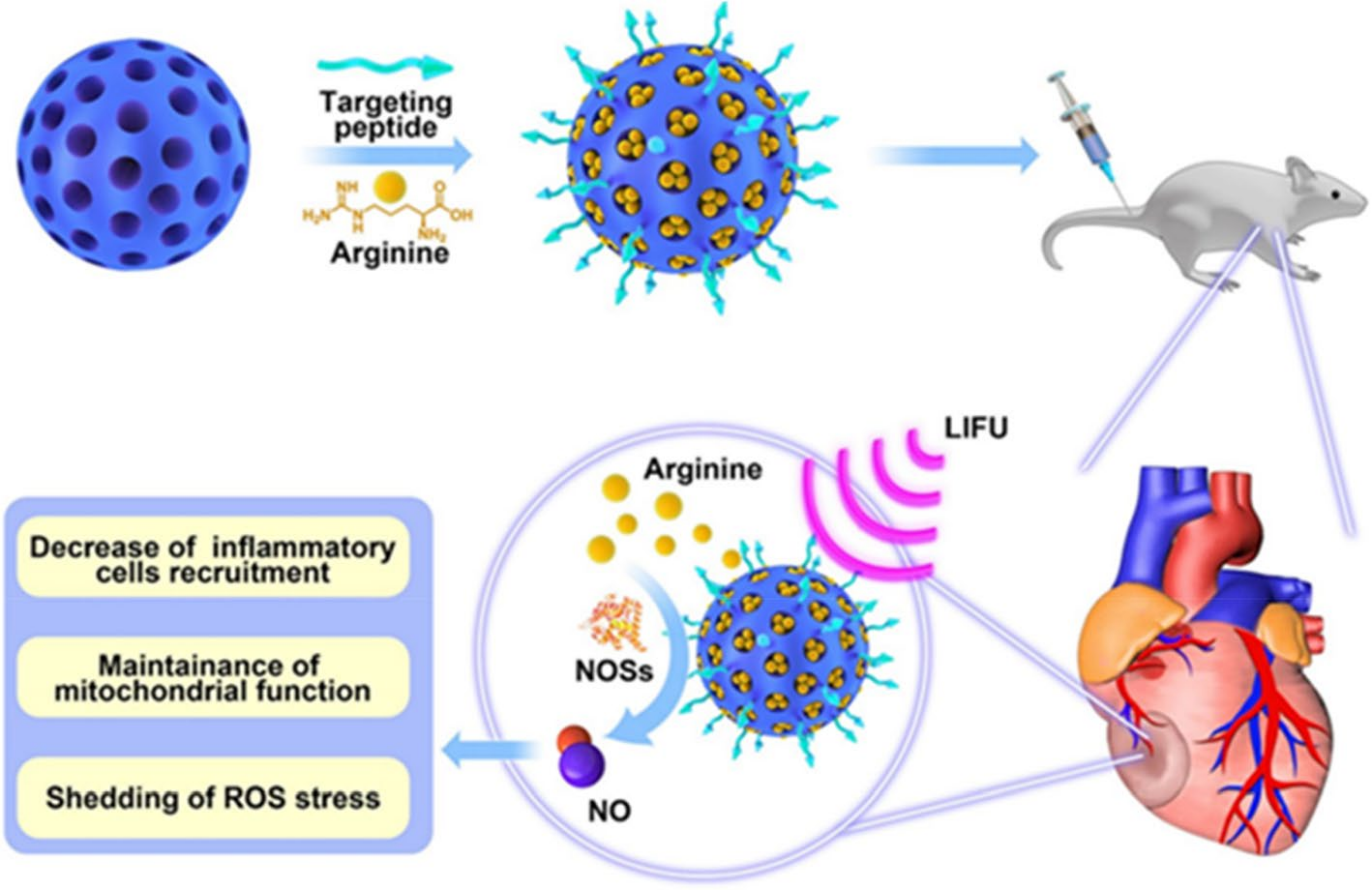
Design of heart-targeting L-arginine loaded mesoporous silica nanoparticles (PCM-MSN@LA) and its combined application with low-intensity focused ultrasound (LIFU) to prevent cardiac damage in mice [192]

Utilizing a similar approach, Ouyang and coworkers designed hollow mesoporous silica nanoparticles (PCM-MSN@LA) loaded with L-arginine (LA) as nitric oxide (NO)-releasing agent and modified with primary cardiomyocytes specific peptide (PCM) for heart tissues-targeted delivery to combat LPS-induced cardiac injury [192]. As illustrated in Fig. 12, cardiac targeting was further increased by applying low-intensity focused ultrasound (LIFU). PCM-MSN@LA were found to have significantly higher localization and affinity to cardiomyocytes compared to the hepatoblastoma cell line (HepG2) as a control, suggesting PCM's cardiac selectivity. Likewise, when evaluated in vivo, the fluorescently labeled PCM-MSN@LA showed 60-fold higher fluorescence intensity in the hearts of mice compared to PCM-unmodified nanoparticles. In contrast, the other organs (kidney, spleen, lung, and liver) showed markedly less nanoparticles distribution. Moreover, the fluorescence intensity of PCM-MSN@LA in mice’s hearts increased by 7-fold upon application of LIFU. In vitro, PCM-MSN@LA and PCM-MSN@LA + LIFU improved the cell viability of LPS-treated cardiomyocytes from 62 to 72% and 80%, respectively. Furthermore, PCM-MSN@LA combined with LIFU significantly reduced inflammatory cells recruitment, mitochondrial dysfunction, and oxidative stress in LPS-induced septic mice's cardiac tissues, thus prevented myocardial injury and cardiac dysfunction. We believe their strategy is promising and holds great potential for preventing sepsis-induced cardiac dysfunction in clinical settings.

Ultimately, the findings of these two studies by Huang et al. and Ouyang et al. show the potential of organs targeting peptides as surface modifiers of nanosystems loaded with cytoprotective agents to prevent organ damage and dysfunction associated with sepsis. However, more avenues are available to explore targeting of other vulnerable organs such as liver, spleen, and lung.

##### Bacterial cells targeting peptides

Beyond targeting specific human body organs and inflammation sites, nanosystems can be tailored to selectively target the causative bacteria, addressing sepsis at the early levels of the pathogen invasion. It is well demonstrated that short peptides possessing cationic or amphiphilic properties can be adsorbed to the negatively charged bacterial membranes, specifically targeting bacterial cells and surmounting cellular barriers [201–203]. However, only one study by Lee et al. has been retrieved about using peptides for nanoparticles surface modification to improve bacterial cell affinity for sepsis management [194]. They utilized the chiral dipeptide D-/L-Cys-Phe (CF) for surface modification of gold nano-bipyramids (GBPs) to improve adsorption to and targeting of bacterial cells.

**Fig. 13:**
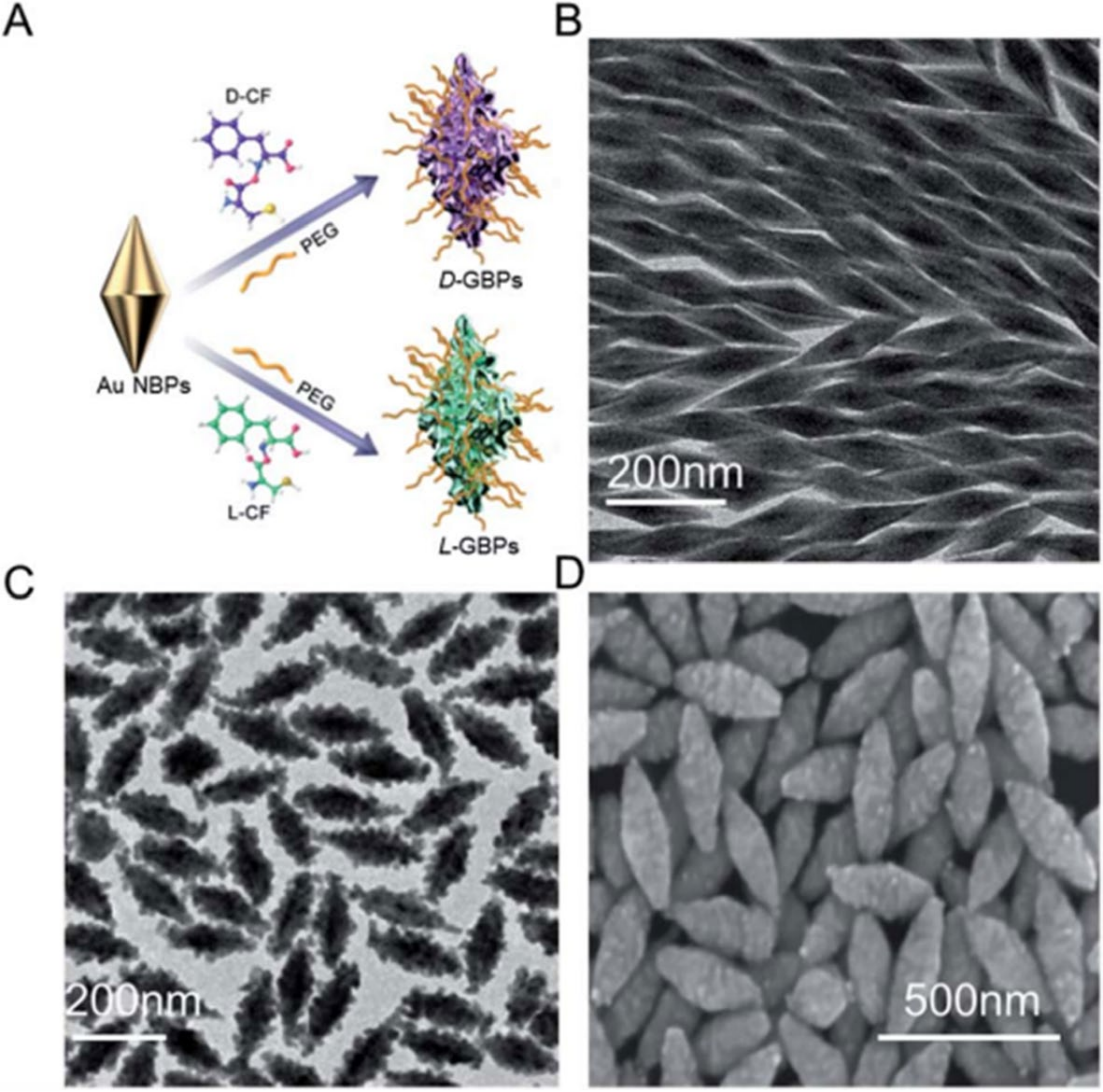
Preparation and characterization of D-/L-GBPs. **A** Coating of GBPs with D-/L-CF (**B**) TEM images of peptide-unmodified Au NBPs (**C**) TEM and (**D**) SEM images of D-/L-GBPs (adopted from ([194])

As depicted in Fig. 13, D-/L-CF peptides gave the GBPs a spike shape (sea cucumber-like morphology) with higher binding affinity to protein A of the *S. aureus cell* membrane. Fluorescence imaging showed a higher overlapping of D-GBPs with the fluorescence of *S. aureus* compared to L-GBPs and DL-GBPs, indicating the higher adsorption and interaction of D-GBPs with bacterial cells. D-/L-GBPs demonstrated good photothermal properties and efficient absorption of near-infrared light irradiation (NIR), raising temperatures above the human body, suggesting they could damage the bacterial cell wall and achieve photothermal antibacterial treatments. Both D-GBPs and L-GBPs showed superior antibacterial activity against *S. aureus* compared to the peptide-unmodified GBPs, with increased activity upon NIR irradiation. Notably, the activity of D-GBPs was substantially higher compared to that of L-GBPs. Evaluated in vivo using *S. aureus*-induced mice sepsis model, D-/L-GBPs treatment showed significant reduction in organs’ bacterial counts and recovery of serum white blood cells levels and animal weights, with the D-GBPs + NIR group being the most effective. It is worth mentioning that their findings prove the strong potential of peptides for coating metallic nanoparticles to achieve bacterial targeting and better antibacterial efficacy. Moreover, their findings shed light on the difference in activity between D- and L-chiral isomers of peptides, which we believe is of significant importance when designing peptides for different purposes.

In summary, the studies highlighted in this section contribute valuable insights into applying peptides as targeting moieties modified on the surface of nanoparticles for effective sepsis management. Three biological targets have been utilized, viz. inflammation sites, specific organs, and bacterial cells. With inflammation sites targeting being the most studied strategy, more avenues are available to unlock the full potential of peptides as targeting motifs for nanoparticles against sepsis, especially bacterial cells targeting.

##### Peptides as nanocarriers for antisepsis agents

Peptides can be designed to have varied surface charges, solubility, and other physicochemical properties that allow them to form stable nanosystems with excellent encapsulation and cargo delivery efficacy [204]. Therefore, Peptides could be used as nanocarriers to improve the antisepsis drugs' stability, cellular uptake, and therapeutic outcomes. Besides, the nanocarrier peptides can be designed to have adjuvant pharmacological activity toward sepsis management, such as antimicrobial and anti-inflammatory efficacy. So far, only three studies [205–207] have been reported on using peptides as nanocarriers for antisepsis agents, highlighting the potential for further exploration to leverage these excellent biomaterials for enhanced and targeted drug delivery against bacterial sepsis. This section will discuss the application of peptides as nanocarriers for antisepsis agents based on the therapeutic effect of the loaded agent, whether it was an anti-inflammatory or antibacterial effect.

As for this, He and co-authors designed polypeptide-based hybrid nanoparticles (HNPs) encapsulating the anti-inflammatory TNF-α small interfering RNA (TNF-α siRNA) to provide endosomal escape and cellular internalization against hepatic sepsis [205]. HNPs were designed from a combination of the cationic helical polypeptide (PPABLG) and the anionic polypeptide (PAOBLG-MPA). The helical structure of PPABLG is supposed to form pores on the cell membrane and provide strong membrane permeability, enhancing the TNF-α siRNA internalization. This intracellular delivery of TNF-α siRNA was evaluated in vitro in LPS-stimulated murine macrophages (RAW 264.7) cells, and the HNPs were found to markedly increase the cellular uptake of TNF-α siRNA, achieving 90% inhibition of TNF-α production, unlike the free TNF-α siRNA which showed a negligible gene-silencing effect. Moreover, HNPs mediated endosomal escape and significantly reduced the colocalization of TNF-α siRNA with the fluorescently labeled endosomes/lysosomes compared to free TNF-α siRNA. When evaluated in an LPS/D-galactosamine (D-GalN)-mice model of hepatic sepsis, the free TNF-α siRNA degraded within 2 hours while the HNPs-encapsulated siRNA remained stable and attained higher accumulation in the macrophage-rich organs such as liver, lung, and spleen. Furthermore, compared to the free siRNA, the HNPs demonstrated a stronger anti-inflammatory efficacy and significantly downregulated TNF-α, rescuing the mice from hepatic sepsis.

While in the previous study, a peptide with cell penetration enhancing properties was used as a nanocarrier for the anti-inflammatory agent, Chen et al. employed an antimicrobial peptide nanogel as a nanocarrier for the anti-inflammatory agent, TNF-Related Apoptosis-Inducing Ligand (TRAIL), for effective sepsis management [206]. The bactericidal cationic poly(L-lysine)-block-poly(L-threonine) co-polypeptide (PLL-b-PLT) was crosslinked to nanogel encapsulating TRAIL protein to accomplish dual antibacterial and anti-inflammatory efficacy to combat bacterial sepsis. The optimized TRAIL nanogel was found to have a spherical shape with size, PDI, ZP, and EE% of 295.3 ± 5.7 nm, 0.21 ± 0.01, 18.5 ± 2.1 mV, and 98.5 ± 2.1%, respectively. Evaluated in MLE-12 (mouse lung epithelial cell) and Raw264.7 (macrophage) cells, TRAIL nanogel exhibited a good biosafety profile against normal cells. However, it showed superior cytotoxicity and apoptosis induction to LPS-activated cells compared to free TRAIL and blank nanogel. Furthermore, TEM analyses of TRAIL nanogel-treated *K. pneumoniae* revealed significant cell membrane destruction and loss of integrity. In vivo, TRAIL-encapsulated nanogel significantly reduced TNF-α and IL-6 levels, blood bacterial load, and pulmonary leukocytes accumulation; thus, it protected mice against *K. pneumoniae-*induced sepsis and LPS-induced lung and kidney injury and prolonged their survival rates. It is noteworthy that their dual therapeutic approach holds great potential in targeting both infecting bacteria and the inflammatory syndrome of sepsis.

In contrast to the other studies in which anti-inflammatory agents have been loaded in peptides-based nanocarriers, Z. Chen and coworkers have encapsulated the antibacterial antisense oligonucleotides (ASOs) in dendritic polypeptides nanoparticles coated with DSPE-mPEG2000 (DP-AD7) against multidrug-resistant bacterial infections and sepsis [207]. The high molecular weight and hydrophilicity of ASOs hinder them from cellular penetration. However, their encapsulation into cationic nanoparticles can provide a solution [208]. While the free ASO could not penetrate bacterial cells, DP-AD7 achieved 90% uptake by *S. aureus*, MRSA, *E. coli*, and extended-spectrum beta-lactamases producing (ESBLs)-*E. coli*. As a result, DP-AD7 significantly inhibited the in vitro growth and the expression of target genes in these bacteria. Going further, when evaluated in septic mice model of ESBLs-*E. coli,* DP-AD7 significantly improved the survival rate and decreased the organs’ bacterial load.

Notably, the findings of the studies in this section underscore peptides as promising nanocarriers for antisepsis agents, including both anti-inflammatory and antibacterial agents. However, none of the discussed studies reported the *evaluation of in vitro drug release*. We believe it would have been worthwhile to investigate the release profiles as they are critical for the clinical use of the nanosystems in terms of dosing frequency and therapeutic efficacy. Overall, peptides exhibit the potential to overcome the inherent limitations that hinder the clinical translation of antisepsis agents, such as improving their stability and cellular internalization.

## Conclusion, challenges and future perspectives

Peptides have been effectively utilized in nanotools against sepsis and have shown enormous potential to improve its diagnosis and management. This review highlighted and critically discussed various reports on using peptides in nanotechnology against sepsis with the capacity to attain prompt diagnosis and efficient management. Based on the reported findings, peptides demonstrated specific and robust interactions with bacterial cell membranes and inflamed tissues, enhancing the bacterial recognition in blood for diagnosis, cell penetration for intracellular nanosystems-payload delivery, membrane disruption for effective bacterial killing, and inflammation-targeted and organ-specific release of nano-delivered antisepsis agents. In addition, engineering within nanosystems significantly overcame the limitation of bioactive peptides (AMPs and AIPs) and enhanced their stability, biosafety, and efficacy. Moreover, peptides have been effectively applied as nanocarriers for antisepsis agents, improving their effectiveness, reducing systemic toxicity, and improving management outcomes. The potentiality of the reviewed nanosystems has been investigated both in vitro and in vivo and found to be superior to that of free bioactive peptides and the peptides-unmodified nanosystems. Cumulatively, these findings reveal diverse and prominent roles peptides can undertake as active agents or excipients in nanosystems for fighting sepsis.

For sepsis diagnosis, it is apparent that peptides were predominantly used as pathogen recognition moieties conjugated to nanoplatforms, focusing mainly on Gram-positive bacteria identification. Thus, more research is recommended to explore using peptides to capture other virulent bacteria that significantly contribute to sepsis development, such as *E. coli* and *K. pneumoniae* [209]. Furthermore, the advancement of molecular sciences and increased understanding of sepsis pathophysiology continue to identify new biomarkers suited for sepsis diagnosis. Henceforth, further investigations to design peptides with high affinity to such biomarkers and engineering them within nanotools could significantly help to advance sepsis diagnosis techniques.

With respect to sepsis management, peptides have been mostly utilized as bioactive peptides that are nano-delivered through self-assembly, encapsulation, or conjugation to surfaces of nanoparticles. With self-assembly and nanoencapsulation being the prevalent explored strategies, linking bioactive peptides to the surface of metallic or organic nanoparticles awaits more investigations as a nano-delivery approach against sepsis. It is perceivable that the application of peptides as targeting moieties on nanosystems has been mostly directed to ICAM-1 targeting to achieve drug release at the inflamed tissues. However, with the development in identifying new proteins and receptors involved in sepsis pathogenesis, peptides could be more investigated to specifically target those proteins such as PAR-1, CD44, and TREM-1 [186], accomplishing improved management outcomes with less systemic toxicity. Moreover, peptides utilization as nanocarriers for antisepsis drugs was the least studied application in nanotechnology for sepsis management. This fosters opportunities for further utilization of these unique biomaterials to enhance antisepsis drug delivery and improve patients’ management outcomes. Finally, with the aid of molecular dynamics simulation and artificial intelligence, peptide sequences can be harnessed to have excellent and improved properties, such as having a stable nano-assembly and strong binding to biological targets and sepsis biomarkers, reducing the cost and accelerating the clinical translation to improve both diagnosis and management of sepsis.

Even with encouraging advancements in the utilization of peptides in sepsis diagnosis and management nanotools, the field is still emerging, and various challenges are encountering that hinder clinical translation. Primarily, these nanoplatforms' design and efficacy depend on the sepsis pathophysiology, which is very discrepant during different disease stages and from patient to patient. Moreover, all the discussed nanosystems have been evaluated in vitro or in vivo on mice models from which human sepsis pathogenesis, prognosis, and treatment response may vary significantly. Therefore, new sepsis models that mimic human responses and outcomes are of great importance in hastening the clinical adoption of the developed nanotools. Moreover, the fabrication of these nanoplatforms is complex, resulting in concerns regarding their reproducibility, scalability, stability, and cost-effectiveness. Additionally, the absence of official quality control guidance and recommendations for nanoplatforms characterization is further delaying their clinical utilization. Thus, optimization and comprehensive evaluation during development are critical to ensure reproducible and scalable manufacturing. Also, pharmacoeconomic and regulatory assessments are required to evaluate the cost-effectiveness of further clinical application of these nanosystems.

Even so, with the ongoing research and in-depth investigations, we envisage that the aforementioned challenges would be overcome and allow for clinical utilization of peptides-based nanosystems to enhance sepsis diagnosis and management practices, ultimately improving patient outcomes. Consequently, this review provides a foundation and comprehensive background for both academia and industry to advance and scale-up these nanoplatforms for efficient sepsis diagnosis and management. To bring it all together, peptides have shown promising potential as active principals and excipients in nanotools against sepsis, achieving rapid identification and on-time superior interventions to ensure better patient outcomes.

## Data Availability

Not applicable.
